# Designing synthetic networks *in silico*: a generalised evolutionary algorithm approach

**DOI:** 10.1186/s12918-017-0499-9

**Published:** 2017-12-02

**Authors:** Robert W. Smith, Bob van Sluijs, Christian Fleck

**Affiliations:** 1 Laboratory of Systems & Synthetic Biology, Wageningen UR, PO Box 8033, Wageningen, 6700EJ The Netherlands; 2 LifeGlimmer GmbH, Markelstrasse 38, Berlin, 12163 Germany

**Keywords:** Evolutionary algorithm, Synthetic biology, Network design

## Abstract

**Background:**

Evolution has led to the development of biological networks that are shaped by environmental signals. Elucidating, understanding and then reconstructing important network motifs is one of the principal aims of Systems & Synthetic Biology. Consequently, previous research has focused on finding optimal network structures and reaction rates that respond to pulses or produce stable oscillations. In this work we present a generalised *in silico* evolutionary algorithm that simultaneously finds network structures and reaction rates (genotypes) that can satisfy multiple defined objectives (phenotypes).

**Results:**

The key step to our approach is to translate a schema/binary-based description of biological networks into systems of ordinary differential equations (ODEs). The ODEs can then be solved numerically to provide dynamic information about an evolved networks functionality. Initially we benchmark algorithm performance by finding optimal networks that can recapitulate concentration time-series data and perform parameter optimisation on oscillatory dynamics of the Repressilator. We go on to show the utility of our algorithm by finding new designs for robust synthetic oscillators, and by performing multi-objective optimisation to find a set of oscillators and feed-forward loops that are optimal at balancing different system properties. In sum, our results not only confirm and build on previous observations but we also provide new designs of synthetic oscillators for experimental construction.

**Conclusions:**

In this work we have presented and tested an evolutionary algorithm that can design a biological network to produce desired output. Given that previous designs of synthetic networks have been limited to subregions of network- and parameter-space, the use of our evolutionary optimisation algorithm will enable Synthetic Biologists to construct new systems with the potential to display a wider range of complex responses.

**Electronic supplementary material:**

The online version of this article (doi:10.1186/s12918-017-0499-9) contains supplementary material, which is available to authorized users.

## Background

Through the efforts of Systems and Synthetic Biologists, we have come to understand that responses of large, complex biological networks are mediated by a series of smaller, interconnected modules or motifs [1, 2]. In combination with synthetic implementation of these network motifs, mathematical modelling has aided the design and exploration of system properties [3–5]. A classic example of the ‘forward engineering’ approach is the Repressilator [3]. Elowitz & Leibler constructed the Repressilator motif in *E. coli* and, using a mathematical model, found that tuning the promoter strength and the protein lifetimes within their plasmid constructs enhanced the likelihood of obtaining oscillations [3]. These initial findings were extended by Tsai et al. who mathematically analysed different Represillator-based network structures and parameter sets, finding that strong auto-regulation of a single Repressilator component enhances the robustness of oscillations [6]. More recently, Potvin-Trottier et al. have improved the performance of the Repressilator experimentally by reducing the effects of noise on the system [7]. Similar work has been performed with toggle switches and feed-forward loops, providing us with a range of modular networks that can reliably produce different responses [4, 5].

Whilst the forward engineering approach has proven highly successful, the opposite challenge (‘reverse engineering’ a network design from a known desired response) is also of importance. Notably this would allow one to obtain novel network designs that may possess complex functionality. In terms of network design, there are two levels that need to be explored [8, 9]. The first level to be explored is the ‘network space’ where all possible network topologies exist. One method commonly used to optimise the topology of promoter circuits is Mixed Integer Non-Linear Programming, a minimisation optimisation routine where parameters can be altered within certain ranges [10–12]. This method has been extended to optimise networks for multiple objectives, resulting in a Pareto front that allows one to observe the trade-offs between different system constraints [10]. The second level is the ‘parameter space’ that contains the reaction rates for a given network topology. Importantly for synthetic network design, recent focus has been to find parameter sets that are robust to stochastic fluctuations thereby increasing the likelihood of successful experimental implementation [7, 13–15]. However, efficient means of executing and solving the reverse engineering problem have yet to be developed in a generalised manner for the synthetic biology community.

A family of techniques that are currently garnering attention for network design are Evolutionary Algorithms (EAs; for reviews see [16, 17]). These methods, in principle, have three advantages over conventional optimisation techniques for synthetic network design:

1. The ‘design space’ — consisting of both the network and parameter spaces — is efficiently explored to find the systems that are able to generate a desired phenotype.
2. One can track the effects of random system perturbations over the course of cellular evolution, much in the same way as observed during laboratory evolution experiments.
3. From the resulting evolutionary trajectories, one may be able to understand how simple motifs have naturally evolved into the much larger and complex networks we observe today in biological systems.

Note that the ‘design space’ is highly multi-dimensional and unbounded implying that a number of different systems can be found that yield the same phenotype. Thus, through the use of EAs, one is left with a number of locally optimal networks to test and validate experimentally. A review of different EAs has shown that they are able to find optimal networks from synthetic datasets [18]. The principles of EAs are in accordance with steps seen in evolution: starting from an initial population, the fitness of the individuals is assessed against some criteria, reproduction (either sexual or asexual) takes place to produce offspring networks that then undergo random mutations before a subsection of the individuals in the population are removed, undergoing the same cycle until some termination criteria are met [19]. Furthermore, these optimisation routines can be extended to incorporate multiple objectives whereby the resulting population of optimal individuals lies on the Pareto front. Along the Pareto front lies the set of ‘non-dominated’ solutions, i.e. a solution cannot become improved for one performance criterium without becoming weaker in another [19, 20]. Thus, the Pareto front provides us with a suite of functionally unique systems that can be constructed experimentally with different properties.

Small modular networks, such as the Repressilator and feed-forward loops, have proved to be good test-cases for EAs [3, 4]. Francois & Hakim analysed the evolution of bi-stable switches and oscillating motifs using ordinary differential equations (ODEs) and asexual reproduction of networks [21]. They observed that post-translational reactions are important for the generation of oscillating systems [21]. Using a different mathematical representation of reactions, Paladugu et al. also obtained a range of small networks able to produce desired responses whilst noting the computational difficulties of evolving large networks using such a mathematical framework [22]. Consequently, recent studies have concentrated on networks of limited size or of limited reactions to find motifs that robustly produce oscillations or respond to inputs [23–27]. Notably, it has been observed that robustness of an oscillator is related to cooperativity between transcription factors (as determined by Hill coefficients greater than 1) rather than system complexity [26]. By increasing the cooperativity between components the system becomes more non-linear, which is an important design feature for oscillating networks [28]. Futhermore, Rodrigo et al. have tracked the evolution of small oscillating networks to propose how circadian clocks have adapted through evolution [29]. Thus, EAs allow us to understand a broad range of system dynamics and their occurrence in nature.

To alleviate the computational load of searching such a large ‘design space’, different methods of model description have been considered. First, Feng et al. used rule-based modelling whereby all the model information is contained in schema (strings and matrices) to evolve protein signalling networks [30, 31]. Second, Chang et al. have translated the schema into formatted ODEs to evolve synthetic oscillators of different size [32]. One advantage of containing system information in schema is that mutation and sexual reproduction (or crossover) of model pairs can be performed easily [19]. Finally, none of the above cases have taken into account multi-objective optimisation. Recently, Boada et al. have incorporated multi-objective EA steps into their parameter optimisation for a type-1 incoherent feed-forward loop [4, 33]. The resulting Pareto front highlighted how to tune system responses between different desired situations. Consequently, understanding the trade-offs of certain system properties is an important aspect of systems design.

In this work, we introduce a generalised EA to solve the reverse engineering problem for synthetic biology. The EA combines schema representation, which allows for reproduction between networks, with a mathematically-tractable framework that can obtain optimal networks of any size for any desired response. This brings together the ideas of Chang et al. and Feng et al. with those of Francois & Hakim and Paladugu et al. [21, 22, 30, 32]. Using oscillatory systems and feed-forward loops (see Supplementary Information) as test cases, we build on and generalise previous observations to design oscillating systems. Finally, we incorporate multiple objectives (as in Boada et al.) within our network optimisation [33]. To the best of our knowledge such multi-objective optimisation of network topologies (not just reaction rates) has not yet been utilised for biological systems.

## Methods

Here, we shall describe how our EA is encoded and provide details as to the tests performed in this study. The most important concept within the EA is to translate binary strings/schema into reaction schemes for simulation by ODEs. This allows the EA to assess and optimise networks based on dynamic behaviours. In the Additional file 1, interested readers can find extra details about our EA implementation and possible extensions. The Python scripts are available at https://gitlab.com/wurssb/Evolutionary_Algorithm_Network_Design.

### Network structure

Within our EA we describe biological networks at three levels: i) at the node level whereby a binary string determines how a node within the network is regulated and how it functions, ii) an adjacency matrix that shows how the nodes are connected, and iii) a set of parameters that determine the rate of each reaction. Ultimately, for a general network containing *M* components and *N* irreversible reactions (note that reversible reactions can be split into two irreversible reactions), we look to construct a reaction scheme

1 $$ \sum_{i = 1}^{M} R_{ij}X_{i} {\stackrel{k_{j}}{\longrightarrow}} \sum_{i = 1}^{M}P_{ij}X_{i},   $$

where *j*∈ [ 1,*N*]. Thus, **R** and **P** are *N*×*M* matrices representing the number of reactants and products in a reaction, respectively, and **k** is a vector of *N* reaction rates. This is a similar style to that used by [30, 32] and is discussed in [19]. Here, we shall give an overview of how a network is constructed with examples.

#### Individual genes

Each node of the network represents a single gene. By default, each gene contains the fundamental base reactions required to transcribe DNA (no superscript), translate mRNA (superscript *m*) into functional proteins (superscript *p*) that can form dimers (superscript ∗). For example the gene *G* in node *X* has the following reactions by default

2 $$ \begin{aligned} G_{X} {\stackrel{k_{1}}{\longrightarrow}} G_{X} + G_{X}^{m} \ &\text{(Transcription)} \\ G_{X}^{m} {\stackrel{k_{2}}{\longrightarrow}} G_{X}^{p} \ &\text{(Translation)} \\ G_{X}^{p} + G_{X}^{p} \overset{k_{3}}{\underset{k_{4}}{\rightleftharpoons}} G_{X}^{\ast} \ &\text{(Dimerisation)} \\ G_{X}^{m} {\stackrel{k_{5}}{\longrightarrow}} &\text{(mRNA Degradation)} \\ G_{X}^{p} {\stackrel{k_{6}}{\longrightarrow}} &\text{(Protein Degradation)} \\ G_{X}^{\ast} {\stackrel{k_{7}}{\longrightarrow}} &\text{(Dimer Degradation)}. \end{aligned}  $$

**Fig. 1:**
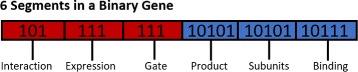
Schematic overview of binary strings describing node regulation and function. Red section denotes the promoter sequence that determines how the node is regulated by a transcription factor (TF, Additional file 1: Table S1). Blue section denotes the function of the resulting protein as determined by the reactions outlined in Additional file 1: Tables S2-S5. Importantly, the order in which these sections appears in the binary string is not important for the function of the EA

To determine how each of the reactions is regulated by other components of the system and how the resulting protein regulates other components in the system, a binary string relates pre-defined regulatory steps to a nodes reaction scheme (Fig. 1). The binary string is divided up into sections each representing a different part of the genes regulation or function. For example, in Fig. 1, the red sections refer to how a node is regulated by other nodes, i.e. whether a transcription factor represses or activates transcription (‘Interaction’), whether the gene is constitutively expressed or not (‘Expression’), and how the transcription factor binds to the promoter (‘Gate’). The blue regions of Fig. 1 determine how the resulting protein functions, i.e. the protein is a transcription factor (‘Product’), or made of multiple subunits (‘Subunits’), or forms higher level complexes (‘Binding’). These regions are ultimately important when one considers how nodes interact on a network level.

Given the different regulatory and/or functional options each gene can have, the EA takes the string sections from the binary string and compares them to dictionaries that have to be pre-defined at the start of the EA. For example, in Fig. 1, the second subsection (‘Expression’) determines whether the genes mRNA is regulated consitutively or requires a TF via logic-gate regulation. This string is of length 3 bits, thus there are 2^3^=8 different possible strings. These 8 strings are randomly divided over the two possibilities based on probabilities defined by the user at the start of each EA run, i.e.

$$\begin{array}{ccc} \mathbf{Expression} & \mathbf{Probability} & \mathbf{Binary strings} \\ \text{Constitutive} & 3/8 & (0,0,0),(1,0,1),(0,0,1) \\ \text{Regulated} & 5/8 & (0,1,0),(1,0,0),(0,1,1),(1,1,0),(1,1,1) \end{array}. $$

Hence, as our example gene contains a (1,1,1) string then we know the mRNA expression is regulated by the transcription factor(s) of other nodes in the network. In our EA implementation, transcription factors can regulate mRNA production through three *effective regulatory gates*: competitive binding between monomeric transcription factors, cofactor regulation whereby multiple transcription factors are required for expression, or competitive binding of protein complexes. In this final scenario, complexes of activating (or inhibiting) transcription factors compete for a single binding region of the target promoter.

These dictionaries are maintained for the rest of the EA. The same idea is applied for all regions of the gene binary string and allows our EA to translate the evolving strings into functional modules.

#### Network description

**Fig. 2:**
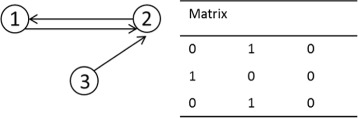
Example illustrating the adjacency matrix for a network. Schematic overview and the adjacency matrix for a given network. Columns of the matrix represent how a node is regulated by other components of the networks. Rows show how a given node regulates other components in the network

To determine how nodes within a network are connected, we use adjacency matrices from graph theory [34]. A single *M*×*M* matrix, **A**, contains all the information about a network (Fig. 2). An element of **A**, *A*_*ij*_, is ‘1’ if a connection between node *i* and *j* exists, and is ‘0’ otherwise. Each row, *A*_*i*∗_, states that node *i* regulates other nodes in the system. Each column, *A*_∗*j*_, details how node *j* is regulated by other nodes in the system. Thus, from the network of Fig. 2, one can see that node 2 is regulated by nodes 1 and 3 (due to the 1’s in entries *A*_12_ and *A*_32_) and goes on to regulate node 1 (due to the 1 in *A*_21_). As we shall see below, this allows for the easy addition, deletion and mutation of network connections during the EA. Note that the adjacency matrix contains no information about the nature of the regulation between nodes.

#### Gene regulation

The EA maps the information contained in the binary genes and the adjacency matrix to a user-defined reaction library (Additional file 1: Figure S1). The reaction library is a list of potential reactions that are sequentially ordered to construct reaction schema (Additional file 1: Tables S2-S6). Each of these reactions is given a unique name (Additional file 1: Figure S2). Ultimately, the ordering of preferred reactions influences the resulting dynamics of the system. To clarify this point more directly we present a simple example.

#### Example #1: gene interactions

From the network of Fig. 2, we can see that gene 3 (*G*_3_) regulates gene 2 (*G*_2_). In our example the genes are described by the following binary strings

3 $$\begin{array}{@{}rcl@{}} \begin{array}{ccccccccccccccc} G: &[&\text{Interaction} &|& \text{Expression} &|& \text{Gate} &|& \text{Product} &|& \text{Subunit} &|& \text{Binding}&]& \\ G_{2}: &[& 101 &|& 111 &|& 111 &|& 10101 &|& 10101 &|& 10101 &]& \\ G_{3}: &[& 100 &|& 011 &|& 101 &|& 01110 &|& 10110 &|& 01101 &]&.  \end{array} \end{array} $$

These strings tell us that *G*_2_ is activated (assuming that 101 refers to transcriptional activation) by *G*_3_ complexes (if 01101 refers to protein-protein binding taking place) and goes on to form a monomeric protein that represses *G*_1_ (assuming 10101 refers to a transcription factor function and 10101 leads to monomeric proteins). Here, we will also assume that *G*_3_ regulates *G*_2_ in an uncompetitive manner with *G*_1_ (see Fig. 2). Now we define the reaction list at the start of the EA as

- Transcription
- Translation
- Protein-Protein Binding
- Gene Activation

(Note that the reaction list can be longer, but if the reaction does not appear in the gene strings then it is ignored — see Additional file 1.) Based on this list we see that interaction of proteins is given precedence over its transcriptional regulation activity. Consequently, we can write down the following reaction scheme for the interaction between *G*_3_ and *G*_2_ (ignoring degradation reactions) using the gene strings defined above:

4 $$ \begin{aligned} G_{2} \rightarrow G_{2} + G_{2}^{m} &\qquad \text{(Transcription)} \\ G_{3} \rightarrow G_{3} + G_{3}^{m} &\qquad \text{(Transcription)} \\ G_{2}^{m} \rightarrow G_{2}^{p} &\qquad \text{(Translation)} \\ G_{3}^{m} \rightarrow G_{3}^{p} &\qquad \text{(Translation)} \\ G_{3}^{p} + G_{3}^{p} \leftrightharpoons G^{\ast}_{3} &\qquad (\text{Dimerisation;}\, \mathrm{given~by}\, G_{3}^{\text{Binding}}\!:\, [\!01101]\!)\\ G_{3}^{\ast} + G_{2} \rightarrow G_{3}^{\ast} + G_{2} + G_{2}^{m} &\qquad (\text{Gene Activation;}\, G_{3}^{\text{Product}}:\, [10110],\\ &\qquad \vphantom{G_{2}^{m^{P}}} G_{2}^{\text{Interaction}}:\, [101]). \end{aligned}  $$

Here, $G_{i}^{\text {f}}:\, [x]$ implies that function f of gene *G*_*i*_ is found by comparing string [*x*] to the reaction dictionaries discussed above — i.e. $G_{3}^{\text {Binding}}:\, [01101]$ tells us that $G_{3}^{p}$ forms a protein complex as [01101] is found in the appropriate reaction dictionary. Now, let’s assume that the pre-defined reaction list is altered such that ‘Gene Activation’ is given precedence over ‘Binding’. This would lead to a different reaction scheme

5 $$\begin{array}{*{20}l} G_{2} \rightarrow G_{2} + G_{2}^{m} \ &\text{(Transcription)}  \\ G_{3} \rightarrow G_{3} + G_{3}^{m} \ &\text{(Transcription)}  \\ G_{2}^{m} \rightarrow G_{2}^{p} \ &\text{(Translation)}  \\ G_{3}^{m} \rightarrow G_{3}^{p} \ &\text{(Translation)}  \\ G_{3}^{p} + G_{2} \rightarrow G_{3}^{p} + G_{2} + G_{2}^{m} \ &\text{(Gene Activation)} \\ G_{3}^{p} + G_{3}^{p} \leftrightharpoons G_{3}^{\ast} \ &\text{(Dimerisation)}  \end{array} $$

that, upon translation in ODEs (see below), would produce different dynamics to Eq. (4). Notably, in Eq. (5), the dimer sequesters the transcription factor rather than helping to produce the mRNA of *G*_2_.

#### Mathematical formulation of reaction networks

In order to simulate the biological network, the reaction scheme developed above is translated into a system of ODEs following general mass action (GMA) kinetics [35, 36]. One can translate the reaction scheme of Eq. (1) into a system of *M* ODEs:

6 $$ \frac{d\vec{\text{X}}}{dt} = \mathbf{S}\cdot\mathbf{v}(\vec{\text{X}}),   $$

where **S**=(**P**−**R**)^*T*^ is the stoichiometry matrix of the system and

$$ v_{j}(\vec{\text{X}}) = k_{j}\prod_{i = 1}^{M}X_{i}^{R_{ij}},   $$

is the flux vector as defined in [36]. The initial conditions are given by $\vec {\text {X}_{0}}$ and can be set in a user-defined manner.

### Evolutionary algorithm

**Fig. 3:**
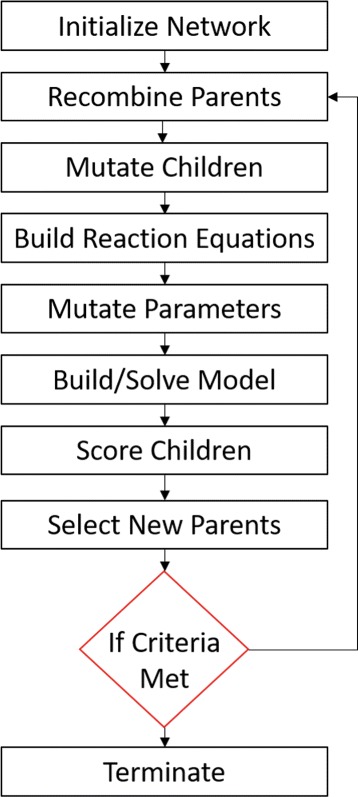
Schematic overview of the evolutionary algorithm. An initial population defined by user-selected inputs is generated. The individuals are simulated and scored to determine their phenotypic fitness. Based on their respective fitnesses, two individuals are selected for recombination to produce offspring networks. These child networks are then randomly mutated and scored for their respective fitnesses. If the child is fitter than the parents then it is kept, otherwise the child is rejected and a new offspring is created. These steps are repeated until pre-defined termination criteria are met

In the following, we shall discuss the concepts of the steps taken during our EA. The schematic outline of the EA can be seen in Fig. 3 and follows steps described in [16, 17, 19]. We endeavour to keep the descriptions as general as possible since many options are user-defined for specific problems of interest.

#### Initialisation

To start the EA, an initial population of individual networks needs to be constructed. The user can define the size of these initial networks or leave this to be randomly chosen from a uniform distribution between the allowed minimum and maximum network size. The maximum network size, defined by the number of gene nodes in the network, can be specified by the user. The initial set of networks are then created randomly by constructing adjacency matrices (with at least 1 connection between nodes) and random schema describing each node. One could also decide that all initial networks are the same by prescribing specific adjacency matrices and node schema.

#### Determining reaction rates

Parameter rates for each reaction within the network can be predefined or are obtained from a user-defined probability distribution *P*(*k*_*j*_). If the parameters of the initial networks are known then the parameter can be set to provide this value. In the mutation step of the algorithm, parameter values can be re-selected from the initial ‘global’ probability distribution, *P*(*k*_*j*_), or from a user-defined local probability distribution that restricts changes in reaction rates to a small region relative to the original value. Additionally, one can define a subset of parameters within the network that do not undergo mutation or, in the event that a new node or connection is created within the network, new parameters are selected from *P*(*k*_*j*_).

#### Selection

Upon the generation of an initial population, the individuals are assessed for their fitness by calculating their respective fitness scores *Δ*. Selection then takes place by ranking the individuals based on these scores. Here we examine three ranking methods [19]:

- *Proportional*: the probability of selection is related to the relative fitness of the individual as compared to all other individuals within the population. Thus, the fittest networks are not always selected as parents.
- *Semi-proportional*: the fittest individual is selected as one parent, and the second parent is selected using a proportional method. This is set as the default option in our EA, as shall be justified in the “Results” section.
- *Elite*: the two fittest individuals are selected for recombination. The use of elitist methods has been shown to increase the speed of convergence towards an acceptable solution [19].

Given the selection of two individuals, these parental networks go on to form child networks via recombination and mutation steps (if a child has a lower fitness than the parental networks then this child is rejected and replaced by a fitter child) that make up the next generation of individuals. Thus, over the generations, the population of networks is consistently increasing in fitness/match to the desired response.

#### Recombination

Recombination between networks takes place by sharing information contained within the adjacency matrices. The recombination process is, essentially, a matrix transformation that combines the adjacency matrices of the two parent networks (**A**_0_ of size *m*×*m* and **A**_1_ of size *n*×*n* with *n*≥*m*) into a child adjacency matrix of size *l*×*l* (*m*≤*l*≤*n*), i.e. $\mathbb {R}^{m \times m} \times \mathbb {R}^{n \times n} \rightarrow \mathbb {R}^{l \times l}$. The size of the child adjacency matrix *l* is chosen from the distribution

7 $$ \mathcal{P}(l) = \frac{2^{n-l}}{2^{n-m+1}-1}, n\geq l \geq m.   $$

Given this distribution, the probability of creating a large child network each generation is lower than the probability of creating small or medium sized networks.

Next, a random binary vector, $\vec {w}$, of length *l* is generated such that

8 $$ w_{i} = \left\{ \begin{array}{ll} p\text{,} & p\in\{0,1\} \,\,\text{with} \,\,\mathcal{P}(p) = \frac{1}{2}, 1 \leq i \leq m \\ 1\text{,} & m < i \leq l\text{.} \end{array} \right.  $$

From this vector we can define $\mathbf {W} = \text {diag}(\vec {w})$ and $\bar {\vec {w}} = \text {diag}(\bar {\vec {w}})$, where $\bar {\vec {w}}$ is the binary complement of $\vec {w}$. Further, we define the matrices **I**_0_ and **I**_1_ to be *l*×*m* and *n*×*l* matrices, respectively, that contain *m*×*m* and *l*×*l* identity matrices augmented with extra *l*−*m* and *n*−*l* zero rows. Finally, we can construct the child adjacency matrix using:

9 $$ \mathbf{A} = \bar{\mathbf{W}}\mathbf{I}_{0}\mathbf{A}_{0}\mathbf{I}_{0}^{\top} + \mathbf{W}\mathbf{I}_{1}^{\top}\mathbf{A}_{1}\mathbf{I}_{1}.   $$

#### Example #2: recombination

To show how recombination works, we provide a brief example. The parent adjacency matrices are given by:

10 $$\begin{array}{*{20}l} \mathbf{A}_{0} &= \left[\begin{array}{cc} 0 & 1 \\ 1 & 0 \end{array}\right]  \\ \mathbf{A}_{1} &= \left[\begin{array}{ccc} 1 & 1 & 0 \\ 1 & 0 & 1 \\ 0 & 1 & 0 \end{array}\right].  \end{array} $$

The size of the resulting child matrix can then be either *l*=2 or *l*=3 with $\mathcal {P}(l=2) = 2/3$ and $\mathcal {P}(l=3) = 1/3$. Let’s assume the child matrix will be of size *l*=3. We then construct $\bar {\vec {w}} = (1,0,1)$ where *w*_1_ and *w*_2_ are randomly drawn and *w*_3_=1 since *l*>*m*. In the case that *l*=2, $\vec {w}$ is of length 2 without the additional 1. Thus, $\bar {\vec {w}} = (0,1,0)$ and

11 $$\begin{array}{*{20}l} \mathbf{A} &= \bar{\mathbf{W}}\mathbf{I}_{0}\mathbf{A}_{0}\mathbf{I}_{0}^{\top} + \mathbf{W}\mathbf{I}_{1}^{\top}\mathbf{A}_{1}\mathbf{I}_{1}  \\ &= \left[\begin{array}{ccc} 0 & 0 & 0 \\ 0 & 1 & 0 \\ 0 & 0 & 0 \end{array}\right] \left[\begin{array}{cc} 1 & 0 \\ 0 & 1 \\ 0 & 0 \end{array}\right] \left[\begin{array}{cc} 0 & 1 \\ 1 & 0 \end{array}\right] \left[\begin{array}{ccc} 1 & 0 & 0 \\ 0 & 1 & 0 \end{array}\right]  \\ &\ \ \ \ \ \ \ \ \ \ + \left[\begin{array}{ccc} 1 & 0 & 0 \\ 0 & 0 & 0 \\ 0 & 0 & 1 \end{array}\right] \left[\begin{array}{ccc} 1 & 0 & 0 \\ 0 & 1 & 0 \\ 0 & 0 & 1 \end{array}\right] \left[\begin{array}{ccc} 1 & 1 & 0 \\ 1 & 0 & 1 \\ 0 & 1 & 0 \end{array}\right] \left[\begin{array}{ccc} 1 & 0 & 0 \\ 0 & 1 & 0 \\ 0 & 0 & 1 \end{array}\right]  \\ &= \left[\begin{array}{ccc} 1 & 1 & 0 \\ 1 & 0 & 0 \\ 0 & 1 & 0 \end{array}\right].  \end{array} $$

Hence, the child matrix is highly reminiscent of **A**_1_ with a missing connection between genes 2 and 3.

#### Mutation

**Fig. 4:**
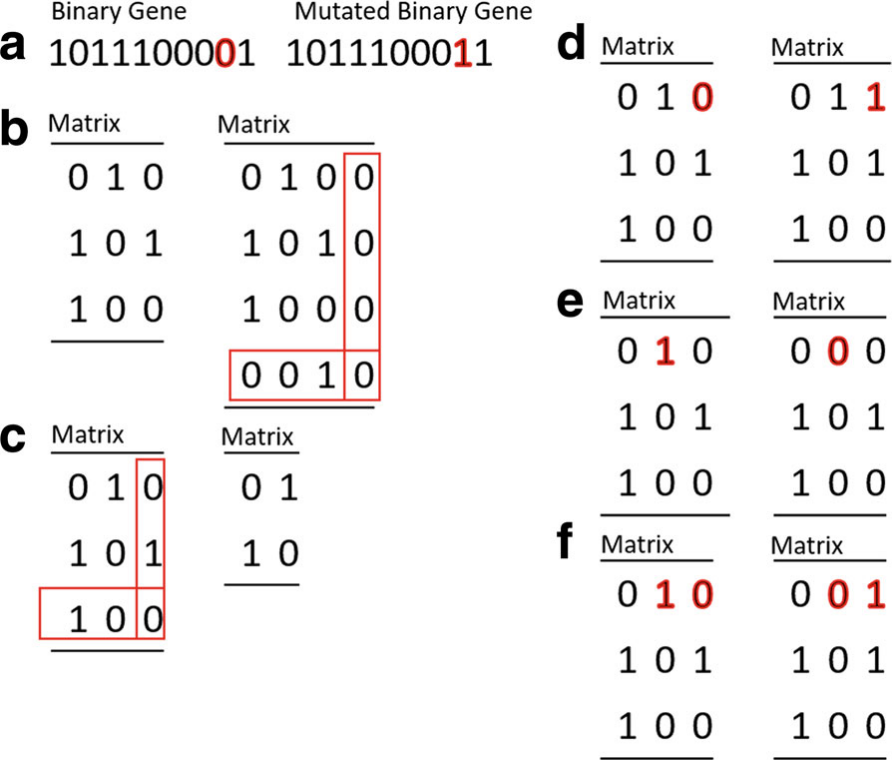
Possible network mutations. **a** Mutating the binary gene: The binary schema of a gene can be mutated by changing a ‘0’ to a ‘1’ (or *vice versa*) in the nodes string. **b**, **c** Adding or deleting nodes: Nodes can be added or deleted by altering the number of columns and rows in the adjacency matrix. New nodes are given random functionality/binary strings. **d**, **e** Adding or deleting connections: Connections within the network can be added or deleted by changing ‘0’s and ‘1’s in the adjacency matrix. **f** Moving connections: Connections can be moved in the network by swapping bits within the adjacency matrix

After recombination, the generated child network is then randomly mutated at both the network and parameter level. The EA contains six potential network mutations (Fig. 4): mutations of a genes function (flipping a bit in the binary string), adding or deleting a gene (adding or deleting a row/column from the adjacency matrix), adding or deleting a connection between genes (flipping a bit in the adjacency matrix), and moving a connection (switching values in the adjacency matrix). Each of these mutations can take place with a given user-defined probability. Upon the selection of a particular network property that is mutated, the binary element of the gene string or adjacency matrix that is ultimately mutated is drawn from a uniform distribution (Fig. 4).

#### Example #2 continued: mutation

Now we have a child matrix **A**, we need to determine whether a network mutation takes place. Let’s say that a connection will be lost and, in particular, the self-regulation of gene 1. Thus

12 $$ \mathbf{A} = \left[\begin{array}{ccc} 1 & 1 & 0 \\ 1 & 0 & 0 \\ 0 & 1 & 0 \end{array}\right] \longrightarrow \left[\begin{array}{ccc} 0 & 1 & 0 \\ 1 & 0 & 0 \\ 0 & 1 & 0 \end{array}\right] = \mathbf{A}^{\ast}.   $$

#### Scoring functions

To evaluate the phenotypic fitness of the individual networks within the EA, we need to compare simulations with a specific phenotype. In the main text we shall discuss the cases of matching concentration profiles, obtaining oscillations using both single- and multi-objective criteria and how oscillating mechanisms can be checked for robustness to internal fluctuations. In the Supplementary Information we investigate feed-forward loops. The scoring functions, *Δ*, for all cases are discussed in detail in the Supplementary Information.

In our EA, we are able to optimise networks both for single and multiple objectives. In the case of single objective optimisation, this implies that the characteristics of model simulations and their comparison to the desired criteria can be aggregated into a single value, $\Delta = \sum _{i=1}^{n} \delta _{i}$, where *δ*_*i*_ are independently-calculated objective scores. In the case of multiple objectives, the comparison between the desired system function and simulations yields a vector of objective scores, $\vec {\Delta } = \{\delta _{1},\delta _{2},...,\delta _{n}\}$. So that two parents can be selected for recombination, we need to reduce this vector of scores to a single number for each network that allows us to more easily compare the performance of different systems. For this purpose, we use a ranking system within our EA. Similar methods of multiple objective scoring have been used previously in evolutionary algorithms that are known to perform well and find results that are optimal for multiple criteria (i.e. the Pareto front) [19, 37].

How the networks are ranked can be determined by the user. Here we will discuss two such methods. Given a set of *N* objective scores for *M* networks, one can obtain a matrix of scores ***Δ***:

13 $$ \boldsymbol{\Delta} = \left[\begin{array}{cccc} \delta_{1,1} & \delta_{2,1} & \hdots & \delta_{N,1} \\ \delta_{1,2} & \delta_{2,2} & \hdots & \delta_{N,2} \\ \vdots & \vdots & \vdots & \vdots \\ \delta_{1,M} & \delta_{2,M} & \hdots & \delta_{N,M} \end{array}\right].  $$

Taking the *L*_*n*_-norm of each column in ***Δ*** yields the vector $\vec {\Delta } = (\|\delta _{1\ast }\|,\|\delta _{2\ast }\|,\ldots,\|\delta _{N\ast }\|)$. By ranking these scores (where 1 is the lowest score for an objective and *M* is the highest score) one obtains $\Xi (\vec {\Delta })$, where min$(\Xi (\vec {\Delta }))$ is the optimal network.

An alternative approach would be to calculate the rank matrix ***Ξ*** based on the scores in ***Δ***:

14 $$ \boldsymbol{\Xi} = \left[\begin{array}{cccc} \xi_{1,1} & \xi_{2,1} & \hdots & \xi_{N,1} \\ \xi_{1,2} & \xi_{2,2} & \hdots & \xi_{N,2} \\ \vdots & \vdots & \vdots & \vdots \\ \xi_{1,M} & \xi_{2,M} & \hdots & \xi_{N,M} \end{array}\right],  $$

with $\vec {\Xi } = (\|\xi _{1\ast }\|,\|\xi _{2\ast }\|,...,\|\xi _{N\ast }\|)$ representing the *L*_*n*_-norm of each column. The optimal network is then obtained from min$(\vec {\Xi })$. Notably, these two methods result in differing results — using $\Xi (\vec {\Delta })$ leads to networks that perform well in one of the *N* objectives being favoured over those that are able to equally balance multiple objectives, obtained from min($\vec {\Xi }$).

#### Settings to obtain presented results

In the examples we present in the “Results” section, we use the following options:

- The initial conditions for each component in each network, $\vec {\text {X}_{0}}$, are set to 0.01.
- Initial networks are either randomly generated or fixed to specific network topologies (e.g. Repressilator or feed-forward loop).
- Parameters are drawn globally from a uniform distribution
  15 $$ k_{j} \sim U\left(k_{j}^{L},k_{j}^{U}\right),   $$
  where $k_{j}^{L}$ and $k_{j}^{U}$ are the lower and upper bounds of the parameter space set in Additional file 1: Table S7, respectively, and;
- Local parameter mutations are found by randomly selecting a new parameter value with 10% deviation from the original value.
- The probability of specific network mutations are set in Additional file 1: Table S8.
- The reaction library available to each network in the EA is discussed in the Supplementary Information.
- Networks are ranked and selected using min$(\vec {\Xi })$ using the *L*_1_-norm.
- In the tests for Fig. 6 d and e, the following user-defined parameters have been randomised: selection method, optimisation objective (i.e. data from Fig. 6 a-c that model is compared with), maximum number of genes allowed in each network, size of network populations, number of allowed network & parameter mutations, parameter mutation method (global or local), probability of gene addition, deletion or mutation, and probability of added, deleted or mutated network connection. In each of the 1933 successful EA runs, each option for the user-defined parameters (e.g. there are three types of selection method possible: proportional, semi-proportional, and elitist) had equal probability of being selected.

#### Termination

We have two termination criteria:

1. If the score of the fittest network has not changed for 250 generations, i.e. the optimisation has converged to a solution, and;
2. The maximum number of generations (2500) has been evaluated without convergence.

Whilst it is possible that the fittest network obtained after 2500 generations is only suboptimal, studies have shown that elitist EAs have the capability to find the global optimum within a finite number of generations [38].

### Programs and solvers

The algorithm is written in Python version 2.7 (Python Software Foundation, www.python.org). Implementation details can be found in the Additional file 1. The source code is available at https://gitlab.com/wurssb. The GMA models are simulated deterministically using ODEINT from the Scipy package or stochastically using the Gillespie algorithm [39]. However, whilst we have provided code to simulate systems using the Gillespie algorithm, note that this greatly increases the computational time of the EA.

## Results

### Benchmarking algorithm performance

Before applying our EA for the purposes of network design, we wished to determine how algorithm performance depends on the user-defined settings and scoring functions. Subsequently, we performed two tests (see Additional file 1). First, we calculated the duration of computational time taken to simulate a generation of oscillating networks of different sizes for 6000 time-points. Second, we randomly selected algorithm parameters for 1933 successful EA runs (see “Methods” section). Based on these results we could determine which algorithm settings had the largest influence on the results and are optimal for use.

**Fig. 5:**
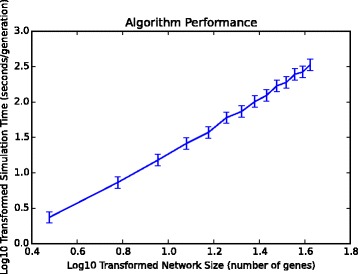
Simulation time of a single generation increases with network size. We ran the EA 25 times for oscillating networks of different sizes *N* (from 3 to 42 nodes). We recorded the average simulation time, *T*, for a single generation. Error bars represent standard deviation. By calculating log_10_(*T*)=*α*log_10_(*N*)+*β* we found *α* = 1.7-1.8 implying that simulation time increases nearly quadratically with network size

As shown in Fig. 5, the computational time required to simulate a single generation of 10 oscillating networks increases with network size. For networks containing 6 nodes, the population can be simulated in under 5 s, whilst approximately 4 min is required for systems consisting of 40 nodes (Fig. 5). This is in accordance with previous observations that performance robustness of gene networks — an important factor for synthetic systems — does not increase in larger networks that maintain oscillations [27].

**Fig. 6:**
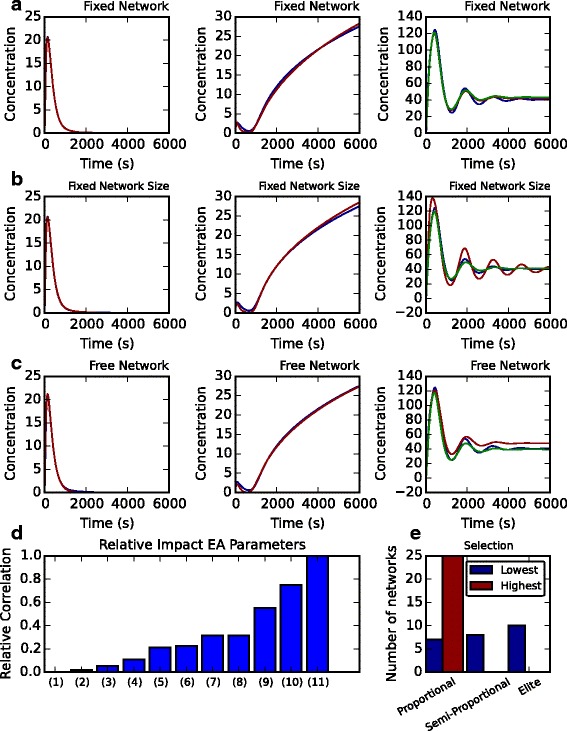
Assessing the influence of different algorithm settings on performance. **a**-**c** Optimal simulations (red lines: after 2500 generations; green lines: after 40000 generations) are compared to the input time-series data (blue lines) for the three objective functions used. (A) Optimisation where network size and connections are fixed. **b** Optimisation where connections between nodes is left free. **c** Optimisation where network size and connections can be chosen freely. **d** Algorithm settings were randomly selected 1933 times and the convergence between initial and final scores was recorded (see Additional file 1). The convergence score for each property was then averaged across all EA simulations and normalised to property 11. Property 1 = ‘Probability of gene addition’; 2 = ‘Network mutation rate’; 3 = ‘Probability of moving a connection’; 4 = ‘Probability of deleting a connection’; 5 = ‘Probability of gene mutation’; 6 = ‘Maximum number of offspring’; 7 = ‘Optimisation objective’; 8 = ‘Parameter mutation method’; 9 = ‘Probability of adding a connection’; 10 = ‘Parameter mutation rate’; 11 = ‘Selection method’. **e** The best and worst performing 25 EA runs were analysed to determine which selection method was used in the EA run

We next set the maximum network size to 10 and used the semi-proportional ranking method to find optimal networks compared to time-series data. We simulated 611 successful runs of the EA for 2500 generations in the cases of parameter optimisation (with a fixed network, Fig. 6 a), parameter and node connection optimisation (a fixed network size, Fig. 6 b) and full network optimisation (Fig. 6 c). In Fig. 6 a we compare the desired time-series simulation of node ‘0’ in each network (blue line) with the resulting simulation from the fittest individual (red line). When fixing the network structure and optimising for parameters, our EA yielded simulations that show a close match with the desired time-series for two of our objectives (left and centre). We observed similar results when increasing the search space to incorporate network connections and network size (Fig. 6 b and c). However, our algorithm struggled to perfectly match more complex time-series behaviour, such as damped oscillations. Upon increasing the maximum number of generations to 40000 we saw a notable improvement in fit (Fig. 6 a—c, right, green line). This suggests that the EA algorithm functions as we desire and is able to find optimal networks given a desired criteria to match within a finite number of generations.

We next wished to determine how the performance of our EA (in terms of finding optimal networks) was effected by the use of different EA settings. To make this analysis as unbiased as possible on user-defined choices, we randomly selected EA settings for each of the 1933 algorithm runs (see “Methods” section) and calculated the convergence ratio of the final score compared to the initial scores using

$$ \Delta_{C} = \frac{\Delta_{\text{final}}}{\Delta_{\text{initial}}},   $$

where *Δ*_final_ and *Δ*_initial_ are the scores *Δ* of the fittest individual in the final and initial generations, respectively. Thus, if the optimal score is much less than the initial network used in optimisation then the convergence ratio will tend to zero, but if the optimisation process does not converge then the ratio will be 1 or greater. By correlating the convergence scores with the presence or value of a particular algorithm property, we found that the selection method (bar 11), had the largest influence on algorithm performance (Fig. 6 d). For example, the 25 EA runs with the highest convergence scores (i.e. the optimisation routine did not function as desired) all employed the proportional selection method (Fig. 6 e). Conversely, the best performing 25 EA runs with the lowest convergence ratio employed a mix of selection criteria with elite selection being slightly favoured. Thus, the convergence ratio is correlated with the presence of proportional selection criteria. Similarly, the rate of parameter mutation (Fig. 6 d, bar 10) and probability of adding network connections (Fig. 1 d, bar 9) also had a strong influence on whether or not the EA converged to an optimal solution.

### Parameter optimisation of Repressilators provides targets for directed evolution studies

**Fig. 7:**
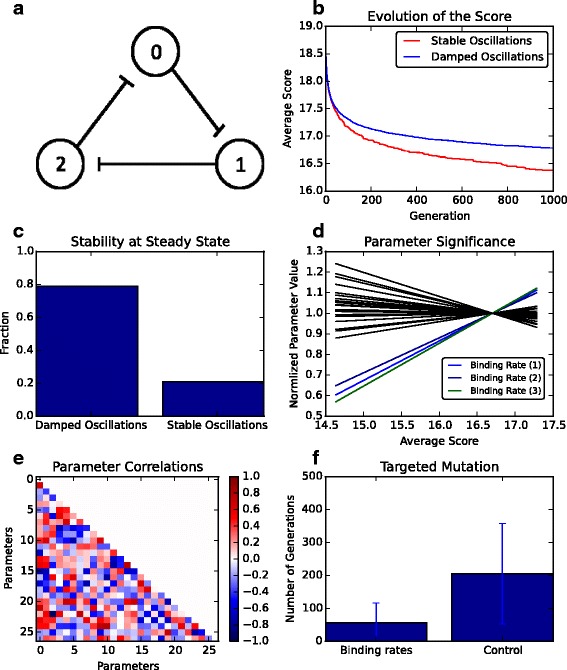
Parameter optimisation of Repressilator systems. **a** Schematic of Repressilator system. **b** Evolution of the average optimal score across generations. Red lines = systems that show limit cycle oscillations over 10 periods, blue lines = systems that show damped oscillations over 10 periods. **c** Percentage of EA runs that produced optimal networks with stable or damped oscillations. **d** Relationship between individual parameters and the objective score (black lines). Selected lines represent the binding rates of Repressilator proteins (blue, dark blue and green lines). **e** Pairwise correlation between system parameters and the objective score (see Additional file 1: Table S9). **f** The number of generations from 272 EA runs until stable oscillations are found when either only the binding rates are evolved or all parameters are allowed to evolve

Using our EA, we next wished to investigate the design of oscillating networks, starting from the synthetic Repressilator system as an example [3]. For details about the scoring function used see the Additional file 1. First, we performed parameter optimisation on the fixed Repressilator network with our EA (Fig. 7 a). We ran our EA successfully 1025 times, and divided the resulting parameter sets into those where stable and damped oscillations could be observed (Fig. 7 b). The presence of oscillations was checked manually by determining whether maxima and minima in the time-series simulations were present and then observing whether these are repeated over the last 10 oscillatory periods of the time-series — if the last 10 periods are identical then the system exhibits stable oscillations, otherwise the system produces damped oscillations. To confirm that our EA can find oscillating networks, we plotted time-series of our optimal network compared to a suboptimal Repressilator (Additional file 1: Figure S3). We found that 20% of the resulting parameters allowed the Repressilator to exhibit stable oscillations (Fig. 7 c).

To determine which parameters have the largest impact on determining whether a system oscillates or not, we plotted the objective score against individual parameter values (Fig. 7 d). This analysis allowed us to determine, based on the steepness of the relationship between parameter value and score, which rates had the largest impact on determining whether a system was more likely to oscillate or not. The results highlight that binding rates between the proteins within the Repressilator are an important factor that determines the presence of oscillations. The importance of post-translational protein cooperativity on oscillating systems has been noted previously [21, 26]. Given the non-linear relationship between components within the Repressilator system, it is likely that combinations of parameters have a larger impact on oscillations than singular parameter perturbations. Further evaluation of the top 25 parameter sets showed that pairs of parameters with highest correlation to the score were production and degradation rates (Fig. 7 e and Additional file 1: Table S9). Thus, tuning production and degradation would influence the presence of oscillations as has been observed in the initial Repressilator design and optimisation studies since [3, 14, 32].

Based on our analysis one could envisage that specifically tuning the protein binding affinities would increase the chance of observing oscillations. By only allowing for the targeted mutation of interaction rates between Repressilator components we determined the number of generations required for the EA to find an oscillating parameter set. As observed in Fig. 7 f, the average number of generations from 272 successful EA runs required to produce oscillating systems is 3.5 times lower when the binding rates are specifically targeted. One could, potentially, test such a scenario experimentally using directed evolution to select for systems with weak interaction rates [40, 41].

### Network optimisation improves objective scores for oscillations

**Fig. 8:**
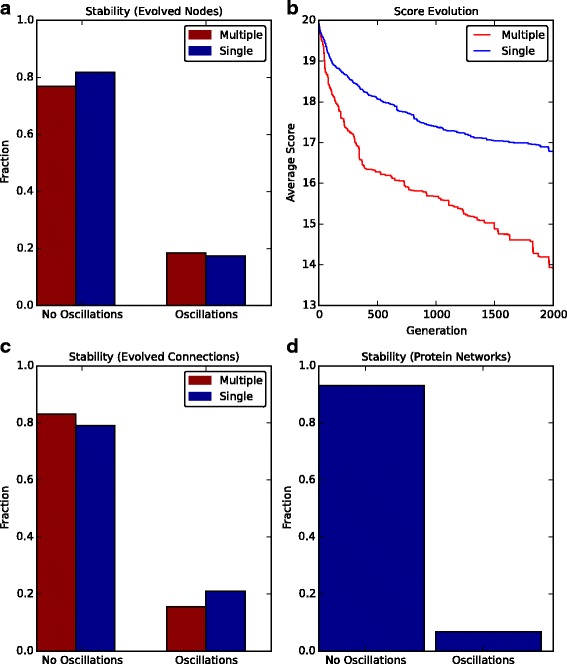
Network optimisation of oscillating systems. **a** Network size is fixed to 4 nodes but connections can be evolved and TF proteins can function as either activators or inhibitors (blue bars) or both (red bars). The final networks are analysed to determine whether they possess limit cycle oscillations. **b** The evolution of the average score across generations from 250 EA runs. Blue line represents evolution of networks with single function TFs, red line represents networks with multi-function TFs. **c** Same as (**a**) except for full network evolution (network size is not fixed). **d** Evolution is performed for post-translational oscillators and networks are analysed for the presence of limit cycle oscillations

We next wished to look whether other networks can be obtained with increased likelihood of oscillations. First, we fixed the network size to 4 nodes and allowed for TFs in the system to either have single (inhibition or activation) or multiple regulatory functions (Fig. 8 a). We found that approximately 15% of the 250 evolved networks had stable limit cycle oscillations from visual inspection (as above). Notably, the likelihood of finding a limit cycle oscillation did not depend on whether TFs had dual functions or not. However, the phenotypic fitness score for systems containing dual-functioning TFs was improved compared to those where TFs function solely as inhibitors or activators (Fig. 8 b).

Interestingly, by removing constraints on network size we obtained similar results with regards to the likelihood of finding an oscillating system with both single- and multi-functioning TFs (Fig. 8 c). However, by comparing how the percentage of oscillating networks depends on network size, one can see that having TFs with multiple functions improves the likelihood of oscillations in larger networks (Additional file 1: Figure S4). Consequently, the likelihood of oscillations does not depend on network size, but on the function of system components, as has been suggested previously [26].

Finally, we looked at the likelihood of oscillating networks being generated by a post-translational network (i.e. there is no gene regulation, Fig. 8 d). A classic example of a post-translational oscillator is the cyanobacterial circadian clock that functions due to phosphorylation cycles [42]. These results show that the probability of finding stable or damped oscillations decreases compared to transcriptional-translation systems. Thus, one could speculate that transcription-translation networks produce more robust oscillations compared to post-translational oscillators, as has been observed previously [43]. One potential underlying cause for this is the increased non-linearities and time-delays maintained within a system that incorporates both transcriptional regulation and post-translational processes rather than protein interactions alone [26, 28].

### Improving the robustness of oscillating networks

**Fig. 9:**
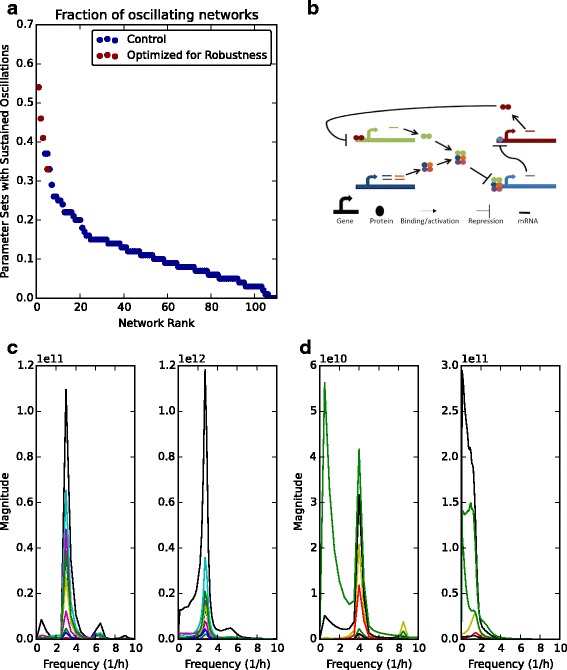
Evolving to obtain robust oscillating networks. **a** Networks obtained from EA runs are scored for robustness (see Additional file 1). Blue dots = networks from Fig. 8 c, red dots = networks obtained when robustness is included as an objective function. **b** Schematic of the network with highest robustness score. **c** Comparison of power spectra obtained after (left) deterministic and (right) stochastic simulations of the network in (**a**). **d** Same as (**c**) for the network with lowest robustness score. Power spectra are the average spectra from (**c**) 2000 and (**d**) 6500 stochastic simulations, respectively

Using the oscillating networks obtained in Fig. 8 c, we checked the systems robustness to parameter perturbations and whether oscillations still occur (see Additional file 1). The networks were then ranked based on the robustness of their system phenotypes (blue dots, Fig. 9 a). As in [27], we define robustness to be the maintenance of system functionality in the face of parameter perturbations. Thus, we calculate robustness as the fraction of networks with parameter perturbations that produce oscillations (see Additional file 1). Interestingly, only ∼ 60 of the networks we obtained from the EA showed the correct oscillatory behaviour for > 10% of the tested parameter sets, whilst less than 20 networks showed oscillations for 20% of the tested parameter perturbations (Fig. 9 a). This suggests that a large number of found networks are very sensitive to perturbations in reaction rates.

To counter this, we wondered whether robustness could be used as an objective for our EA. Due to each parameter of each network needing to be perturbed, the computational time increases dramatically. Thus, we limited our routine to 4 EA runs as a proof of principle (red dots, Fig. 9 a). The networks that were evolved specifically for robustness showed an increase in the fraction of perturbed networks that still maintained oscillatory behaviour. As a comparison of a robust and sensitive oscillating network, we simulated the most robust oscillator (Fig. 9 b) and a sensitive network both deterministically (Additional file 1: Figure S5A) and stochastically using the Gillespie algorithm [39]. The power spectra of the robust network was similar both when calculated from deterministic and stochastic trajectories with a single dominant peak (Fig. 9 c). Notably, within this system, there is not a dominant set of parameter pairs that determine oscillatory behaviour, suggesting that the presence of oscillations may be more likely caused by structural features (Additional file 1: Figure S6). However, for a sensitive system, the dominant peak seen in deterministic simulations is lost when simulated stochastically (Fig. 9 d). This is indicative of internal fluctuations of the network obscuring the underlying low amplitude system dynamics (Additional file 1: Figure S5B).

### Multi-objective analysis provides a set of oscillators with different properties

**Fig. 10:**
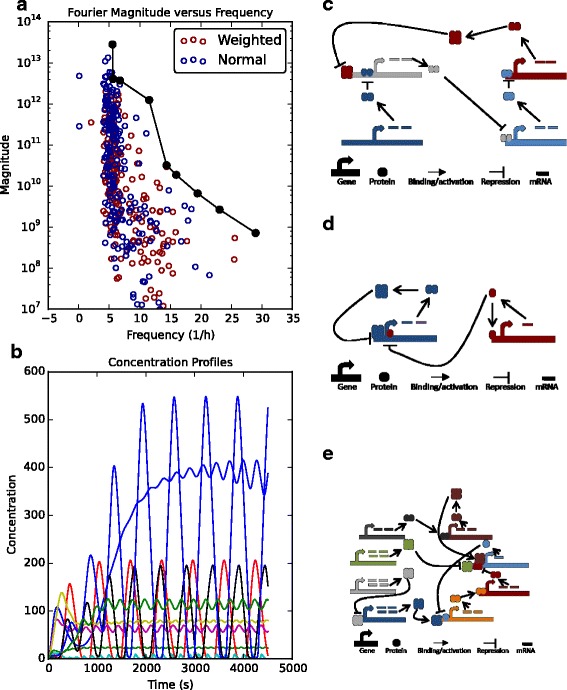
Multi-objective optimisation of oscillating networks for desired amplitudes and periods. **a** The obtained Pareto front of oscillating networks that have optimal amplitude and period properties (black line). Blue dots = optimal systems obtained when objectives have equal weight. Red dots = optimal systems when frequency objective is weighted to double that of other objectives. **b** Deterministic time-series simulations of all networks that lie along the Pareto front. **c**-**e** Schematics of a network with (**c**) high amplitude, low frequency; (**d**) median amplitude and frequency, and; (**e**) low amplitude, high frequency

Whilst optimisation of networks towards a single objective is common in the literature, multi-objective optimisation allows one to look at multiple system properties at the same time [10, 20, 33]. We thus optimised oscillating systems for three properties: high amplitude, higher frequency and minimal width of the resulting power spectra (see Additional file 1). Minimising the width of the power spectra helps to ensure that the resulting oscillations are not a complex combination of multiple sine functions. The amplitude and frequency properties of the resulting optimal networks are shown in Fig. 10 a (blue dots). From this, we see that oscillators generating rhythms of the same frequency can have a range of different amplitudes and *vice versa*. This suggests that amplitude and frequency can be tuned independently of one another. Interestingly, weighting the objectives to favour higher frequencies (that was weighted twice as much as the other two scoring criteria; red dots in Fig. 10 a) marginally increased the chance of obtaining higher frequency oscillators. To illustrate how one can obtain a Pareto front from the multi-objective optimisation, we draw the Pareto front of oscillating networks in Fig. 10 a (black line). Along this line, the optimal networks cannot obtain a higher amplitude without a decrease in frequency or a higher frequency without a smaller amplitude. Time-series concentration profiles of the networks along the Pareto front are shown in Fig. 10 b and, as expected, each network has a unique period length and amplitude.

In Fig. 10 c—e, we highlight the network structures that lie along the Pareto front (a Python script to simulate these systems can be found in Additional file 2). Interestingly, each of these designs are extensions of minimal motifs that have been studied previously. First, the optimal structure for high amplitude, short frequency oscillations is a small extension to the Repressilator system (Fig. 10 c). Second, to generate an oscillation of medium amplitude and frequency, a network consisting of coupled positive and negative feedbacks is Pareto optimal (Fig. 10 d). This network has been studied previously in the context of bi-stable motifs to determine how feedback strength can lead to systems switching between steady-states [44]. Finally, in Fig. 10 e, the Pareto optimal solution for low amplitude and long frequency oscillations is, to the best of our knowledge, a novel network design built upon a negative feedback loop.

### Feed-forward loops have distinct responses to pulsed inputs

**Fig. 11:**
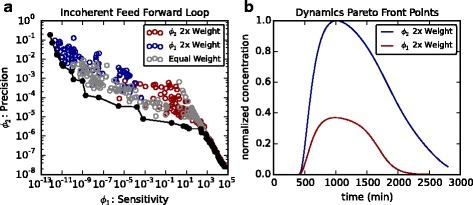
Pareto front for incoherent feed-forward loops as in [33]. **a** Pareto front obtained by weighting different scoring functions. Grey dots = both objectives are equally weighted. Blue dots = sensitivity objective is weighted to double that of the precision objective. Red dots = precision objective is weighted to double that of the sensitivity objective. **b** Simulations from Pareto optimal networks. Blue line is simulation of network optimised for sensitivity to inputs. Red line is simulation from a network optimised for a precise return to pre-input concentration levels

To highlight the utility and generality of our multi-objective EA, we have also analysed the family of feed-forward loops (FFLs; see Additional file 1) [4, 33]. Previous analysis had focussed on one specific member of the FFL family (the type-1 incoherent FFL with AND-gate logic). For this motif we obtained a similar Pareto front to previous publications whereby the network motif can be tuned to either respond maximally to input signals (sensitivity) or show the correct relaxation response back to pre-input conditions (precision; Fig. 11 a) [33]. This, again, confirms that our EA produces results that are consistent with previous observations. Furthermore, by weighting one objective to be twice as influential as the other, we obtained solutions clustered at either end of the Pareto front. Simulations of optimal time-series confirms the trade-off in feed-forward loop dynamics between sensitive and precise responses (Fig. 11 b).

We next asked whether the analysis can be expanded to incorporate all FFLs by evolving parameters and networks connections, whereby different sets of connections (activation/inhibition) are related to different FFL topologies [4]. Additionally, the types of external inputs into the networks (continuous or pulsed) and the regulatory logic gates (AND- or OR-gate) were varied. Interestingly, the results differ depending on the input signal into the system and the type of logic gate employed within the network (Additional file 1: Figure 7). In the case of continuous inputs, it is hard to observe a relationship between FFL topology and functionality (Additional file 1: Figure S7A, B). However, with pulsed inputs, different FFL topologies are clustered around different regions of the Pareto front, whereby coherent FFL 4 and incoherent FFL 1 possess opposite relationships between system sensitivity and precision (Additional file 1: Figure S7C, D). By analysing this Pareto clustering effect for more motifs, researchers can look to design families of networks with highly specified or tunable functions.

## Discussion

### EA implementation expands on previous strategies

In this work we have presented a generalised multi-objective network optimisation routine based on the principles of EAs for the purpose of designing synthetic systems (Fig. 2). By combining a schema description (Fig. 1) of model nodes with GMA kinetics (Eq 6), the evolving networks and reaction rates can be simply recombined (Fig. 3) and mutated (Fig. 4). The schema structure that we have employed allows one to track each generation of networks and the mutations that take place. Thus, an evolutionary path of a network from an initial design to a system with specific properties can be found. Furthermore, our algorithm can be easily adapted for further biological test cases through editing of the dictionaries that encode and simulate the networks (see Additional file 1). Importantly, our methodology is one of the first to describe recombination of networks using crossover/sexual reproduction, whereas many previous studies have employed asexual network recombination [21, 22, 30, 32]. This allows for a larger region of the search space to be explored by increasing the variability between individuals and across generations, as has been discussed by evolutionary biologists [45].

With this in mind, we analysed the performance of the algorithm given different settings and objective functions (Fig. 6). We found that the choice of selection method and objective function had the largest influence on the EA?s ability to find optimal networks even when other user-defined choices were randomly selected (Fig. 6 d, e). To assess the functionality of our EA, we initially showed that our EA performs well as an optimisation routine and is able to find parameter sets that generate desired time-series dynamics in the test cases (Fig. 6 a). Similar observations are made when we allow the optimisation process to incorporate new network nodes and connections and is likely due to the termination criteria used in this study (Fig. 6 b, c). These results show that our EA functions as expected and that, given a target phenotype to match, the EA finds a network and parameter set that is quantitatively similar to the input data. Thus, as well as being used as an algorithm to design networks (see below), our algorithm can be used to reverse engineer underlying networks given specific datasets. Researchers will, therefore, be able to compare different models that yield the same response and elucidate the key design principles within these systems.

### Designing oscillatory networks with desired properties

Oscillating biological networks have garnered a lot of attention as researchers attempt to understand the important principles behind their emergence and how similar systems can be constructed [3, 14, 21, 25–29, 32]. The underlying design principles of biological oscillations include having an appropriate number of system components with non-linear interactions such that there is a sufficient time delay in the network [28]. A classic example of a synthetic tool that satisfies these criteria is the Repressilator [3]. Initial explorations to optimise this system focussed on tuning production and degradation rates whilst, more recently, sources of noise have been reduced [3, 7, 14, 32].

In our work we have expanded the tuning possibilities of the Repressilator to include all system parameters (Fig. 7, Additional file 1: Figure S3 and Table S9). We subsequently found that, as well as production and degradation rates of components, the binding affinities between proteins in the Repressilator system have a key role to play in the generation of oscillations (Fig. 7 d and e). The importance of protein dynamics on oscillations has been previously noted by [21, 25, 26]. Furthermore, we found that the most robust networks are primarily based on the Repressilator as has been studied previously (Fig. 9) [7, 27]. Thus, by confirming the observations of previous studies, we are confident that our algorithm accurately finds networks with desired properties.

Finally, we introduced, for the first time, a multi-objective analysis of oscillating gene networks. By optimising oscillating networks to produce rhythms of varying amplitude, period and shape, we obtained a Pareto front along which one can find optimal networks for different combinations of amplitude and frequency (Fig. 10). The networks that lie along the Pareto front provide a set of motifs with different responses, thus designers of future synthetic networks can take advantage of this by choosing the system that produces the required set of properties. Notably, we found that some of these optimal networks share core structural motifs with oscillating mechanisms that have been previously studied. For example, the core motif for an oscillator we found to have low frequency and high amplitude is a Repressilator network (Fig. 10 c). Whilst networks that have an average frequency and magnitude can be based on coupled auto-regulatory positive-negative feedback loops (Fig. 10 d). Consequently, one can move within ‘design space’ between the two networks by adding a new component to the system and tuning the parameters to generate shorter frequency oscillations. To show the generality of these steps, we also obtained a general Pareto front containing all feed-forward motifs, highlighting that FFLs form clusters along the Pareto front depending on the conditions being analysed (Additional file 1: Figure S7). This is, thus, a generalised analysis of that presented in [33], who concentrated on one of the eight possible FFL motifs. We envisage that producing Pareto fronts of synthetic networks will allow for an easier overview of potential mechanisms that can be constructed experimentally to achieve desired needs.

### Applications to evolutionary research

As well as for the purposes of network design and reverse engineering synthetic systems, we believe that this approach can also be beneficial for those that are interested in the evolution of biological systems. By analysing the evolution of networks, one may be able to understand how complex systems have arisen to relate changing environments with observable phenotypes [8, 9, 22, 46]. Such an idea has been previously studied in relation to circadian clocks and understanding how a complex mix of transcriptional feedback loops and post-translational networks leads to 24 h rhythms across *taxa* [29]. We believe that our EA provides a general and easy way of tracking the evolution of networks towards a particular objective using the schema structure we have employed. Consequently, one could build evolutionary trees of network motifs (see Supplementary Information [46]). This opens up a number of interesting questions. For example, given an initial auto-regulatory feedback loop, how could multiple circadian clock mechanisms have been reached via evolution? What is the probability of evolution producing a mechanism primarily made up of post-translational mechanisms (as in mammals) compared to clocks formed by transcriptional feedback loops (as in plants) [47, 48]? How many other network motifs can produce the properties observed by circadian clocks in nature? Similar such questions could be posed for other biological examples.

Further to understanding the development of biological systems across evolution, our EA can also be used to simulate laboratory evolution [40, 41]. By optimising the synthetic Repressilator network, we found that protein binding rates are key to producing stable oscillations (Fig. 7 e). Consequently, by targeting these parameters for evolution, we were able to obtain populations of oscillating systems more quickly than when the whole parameter set is evolved. Based on our results using the test case of the Repressilator, one could use directed evolution to influence protein dynamics and obtain oscillations. Recently, Potvin-Trottier et al. have improved the functionality of the Repressilator by reducing sources of noise in the system [7]. Notably, this involved altering the system such that protein dynamics (degradation and binding to the promoter) were less ‘leaky’. By further incorporating control over protein interactions, one could potentially improve the robustness of the Repressilator further.

## Conclusion

In this work we have presented a multi-objective EA for the design of synthetic networks. Our EA brings together and extends upon previously studied network design strategies [10, 21, 22, 30, 32]. We have shown the generality of our approach by using the EA to evolve networks towards concentration profiles, oscillating dynamics and signal responses. Furthermore our EA can aid one in understanding how biological systems have evolved from simple motifs to more complex networks in the face of changing environmental conditions. Hence, we believe that our optimisation strategy is ideal to reverse engineer novel networks that satisfy particular constraints.

## Additional files

Additional file 1 Supplementary Information. Contains: Implementation of EA algorithm; details regarding the simulations tests performed in the main text; mathematical descriptions of the scoring functions used in optimisation; multi-objective analysis of feed-forward loops; supplementary figures & tables. (PDF 614 kb)

Additional file 2 Simulation Code. A Python script that allows for the simulation of models presented in Fig. 10 c, d & e. (PY 7 kb)
