# Supplementary material for: Designing synthetic networks in silico: a generalised evolutionary algorithm approach
Source: BMC Syst Biol. 2017 Dec 2;11:118. doi: 10.1186/s12918-017-0499-9 (PMC5712201; doi:10.1186/s12918-017-0499-9)
Supplement: Supplementary file 1 — Supplementary Information. Contains: Implementation of EA algorithm; details regarding the simulations tests performed in the main text; mathematical descriptions of the scoring functions used in optimisation; multi-objective analysis of feed-forward loops; supplementary figures & tables. (PDF 614 kb) [file 12918_2017_499_MOESM1_ESM.pdf]

Designing Synthetic Networks *in silico*: Supplementary Information

Robert W. Smith[*†], Bob van Sluijs[*], Christian Fleck[*‡]

Contents

1 Implementation of EA algorithm **3**
1.1 Overview 3
1.2 Current dictionaries 5
1.2.1 Reaction library 5
1.2.2 System input definition 7
1.2.3 Example of encoding binary strings 7
1.3 Basic instructions to create a new EA study 8
1.4 Possible extensions and generalisations 9
1.4.1 Reaction library 9
1.4.2 Scoring 10
1.4.3 Solver 10
1.4.4 Evolutionary Tracking 11

2 Simulation Tests Performed in Main Text **12**

3 Scoring Functions **13**
3.1 Concentration profiles 13
3.2 Oscillations 13
3.3 Robustness 14
3.4 Feed-forward loops 15

[*]Laboratory of Systems & Synthetic Biology, Wageningen UR, PO Box 8033, 6700 EJ Wageningen, The Netherlands.
[†]LifeGlimmer GmbH, 12163 Berlin, Germany
[‡]Corresponding author: christian.fleck@wur.nl.

4 Multi-objective analysis of feed-forward networks **17**
4.1 Results 17
4.1.1 Type-1 incoherent feed-forward loops 17
4.1.2 All feed-forward loops 18

3 References **21**

4 Supplementary Tables **22**

5 Supplementary Figures **31**

1 Implementation of EA algorithm

1.1 Overview

The EA (available at https://gitlab.com/wurssb) makes use of the standard toolboxes found in the Anaconda package for Python version 2.7 (www.continuum.io). The files within the algorithm folder are automatically called once the main file (*main.py*) is executed. The inputs for the algorithm are within *main.py*. These contain everything from mutation probabilities to input patterns and can therefore be adjusted in this file by the user. For more complex adjustments, such as scoring functions or reaction equation libraries, see Section 1.4. The layout of the main file is as follows:

- *Store_data*: This function stores all the data output by the algorithm. By default the algorithm stores the final optimised network and its parameters.
- *Algorithm_(name)*: This function calls the EA, requiring the defined options within *main.py* as input. These inputs are:

  pool: This variable contains the distributions with which certain attributes occur in the 'network space' (e.g. whether a protein is a transcriptional regulator or a ubiquitinase, etc.) and are distributed over the binary genes.

  boundaries: The boundaries variable is a dictionary containing the parameter value boundaries for each parameter type. The user can modify these boundaries at their own discretion.

  simulation_time: The overall simulation time used in the solver for each each individual in each generation. The number of integration steps is automatically multiplied by 60 such that each integration step represents 1 second.

  promoter_length, gene_length, subunit_length, basal_length, dimer_length: The number of bits every attribute occupies on the genes — a relatively longer bit size increases the probability that this part of the gene is mutated.

  operon: The sum of all the bit lengths assigned by the previous 5 variables.

  plasmid_number: The number of plasmids that are in the system, In the continuous solver this is the factor with which the transcription rates are multiplied. In the stochastic solver these are the number of identically transcribed genes in the system.

  output_genes: Number of output genes.

  input_genes: Number of input genes.

  min_genes: Minimum number of genes every individual in a generation must have.

max_genes: Maximum number of genes every individual in a generation is allowed to have.

initial_gene_number: Number of initial genes in the first parental networks.

add_gene: Probability of a gene addition occurring within a network ($p \in [0, 1]$).

mutate_gene: Probability of a gene mutation occurring within a network ($p \in [0, 1]$).

add_connection: Probability of a connection being added in a network ($p \in [0, 1]$).

del_connection: Probability of a network connection being deleted ($p \in [0, 1]$).

move_connection: Probability of moving a connection between a network ($p \in [0, 1]$).

del_gene: Probability of deleting a gene in a network ($p \in [0, 1]$).

event_probability: An array that contains all mutational probabilities, i.e. the previous 7 variables.

input_map: A dictionary that contains the information regarding the target of input signals and the input type. The dictionary key is a number that represents the product of the node that is activated. The value is the manner in which an input is applied to this product, either directly or indirectly.

input_type: Classify the input type (only required for networks regulated by an external source, Section 1.3.2).

pulse_size: The parameter value assigned to the method of activation.

input_pattern: A list with alternating numbers representing the time points at which an input is given. If no input is given then the number is turned into 'None'. Every integer in the list represents a simulation time of 1 minute or 60 integration steps.

selection_method: The method by which the individuals are selected in each generation. There are three selection options available in string form: 'Elite', 'Proportional' and 'Semi_Proportional'.

mutation_method: The method by which parameters are mutated. There are three mutation methods available in string form: 'Global', 'Local', and 'Both'.

parameter_mutation: Number of parameter mutations every individual undergoes in a given generation.

network_mutation: Number of network mutations every individual undergoes each generation.

offspring_number: Number of individuals in every generation (default = 10).

max_iteration: Maximum number of generations the EA cycles through before it is stopped.

path: File path where the user wishes to store the results.

output: The output concentration profiles that are scored. This is a list with numbers where every number corresponds to a specific node in the network. You cannot score a node that does not exist, if your minimum number of genes is 2 but the output is related to a third node then the EA will eventually crash.

In *main.py* there is a function called *order*. The *order* function takes the attributes in the pool variable and distributes these over different binary permutations for each regulatory function (see Fig 1). The resulting variable 'order' acts like a master key that can translate the binary genes and the interactions between them in the appropriate reaction equations. Thus, in some high-dimensional cases the *order* dictionary requires a large amount of CPU memory.

1.2 Current dictionaries

1.2.1 Reaction library

The networks that are constructed within our algorithm are based on the central dogma of biology: DNA/promoter → mRNA → protein. Thus, for a gene, G, in network node, X, we construct three differential equations for the promoter G_X, the mRNA G_X^m, and protein G_X^p. Within each reaction there are several options that are available to be implemented within the networks. For example, a promoter could be regulated by cooperative transcription factors (TFs) via an to produce mRNA. The resulting protein could then undergo homo- or hetero-dimerisation reactions to target other proteins for degradation or could become a TF to regulate other nodes within the network (see Main Text). Here we will briefly describe all possible reactions that are currently encoded within our algorithm as default.

- *Promoter regulation (Supplementary Table 2)*: When a node produces a TF, then the TF can act as an activator or an inhibitor to regulate the mRNA production of a connecting node. Within our algorithm, the regulation of mRNA by TFs can take place via one of three *effective regulatory functions*. We define three forms of *effective regulatory functions*: competitive (TFs compete for a single binding site), cofactoring (TFs form complexes to aid regulation on a single binding site), or sign-dependent competition (where TFs of the same sign — activation or inhibition — form complexes that compete for promoter activity). Whether a TF activates or inhibits a promoter is dependent on matching

between the promoter and protein section of the gene string. If the gene string aligns with the promoter domains then activation takes place, if these are not matched then inhibition takes place.

- *Protein dimerisation (Supplementary Table 3)*: The proteins formed by translation have the option to form reversible homo-dimers regardless of their later function.

- *Protein regulation (Supplementary Table 4)*: As well as homo-dimerisation, protein products can also undergo a range of post-translational modifications such as phosphorylation, sequestration and degradation. Within our algorithm, reactions can be encoded for: protein degradation through complex formation between the ubiquitinating protein and the target protein, protein activation (such as via phosphorylation kinases) through complex formation with an activating protein, and protein sequestration through reversible complex formation with interaction partners.

- *Protein switches (Supplementary Table 5)*: External signals, such as chemicals or light, can also influence the activity of proteins. We account for this via reversible reactions that are dependent on the external inputs. Using similar reaction structures, protein transport can also be accounted for.

- *Subunit regulation (Supplementary Table 6)*: Finally, some proteins are formed by subunits translated from different mRNA strands. Our algorithm can account for these reactions through the ordered binding of protein subunits produced through translation of the mRNA strands.

One should note that all transcription reactions, in reality, are dependent on the availability of polymerases, whilst translation depends on the presence of ribosomes. Whilst we have not directly addressed these components here (we assume that these components maintain a constant level throughout simulations), one can extend the reactions to incorporate such processes.

The creation of the reaction library is in *reaction.py*. This function takes as input the graph structure of the network (nodes and connections) with the type of reactions and its interaction partners. This means that any adjustment in the form of a new reaction type that is added needs to be included in the encryption possibilities of the binary genes and incorporated as an option for the graph structure in *graph.py*. By adding the reaction in this structure the user has the ability to distinguish between reaction types once the reaction equations are created (i.e. the graph structure serves as an input into the function responsible for the creation of a reaction library). In order to unpack this set of nodes and connections into differential equations, reactions have the following format

[[substrates], parameter rate ID: input reactions + reaction type, [product]].

For each node in a network a gene is created in the form of string characters 'g0-gi', where i refers to the ith row and column of the network adjacency matrix. The numbering allows the algorithm to distinguish between specific reactions that occur in a network such that they can be passed on to the next generation without actively tracking these reactions in the form of class objects.

1.2.2 System input definition

Our system definition is also able to identify input signals into the network to a protein of a specified node. The input can be defined as either direct or indirect and can refer to either an activation or inhibition reaction (Supplementary Table 4). A direct input signal mediates switching between different conformational states of a node, whilst an indirect signal requires an intermediate inducer molecule (as in e.g. phosphorylation cycles). This inducer molecule is added to the network and is regulated by synthesis and degradation in a similar manner to other network components. To define the duration of the input, the total simulated time of the network is split into blocks of 60 time-steps (as default — this value can be changed). Each block is then defined to provide a given input from the reaction library. For example

Input Type = {1: 'Indirect Inhibition', 2: 'Direct Activation'}
Input Pattern = [2,2,2,–,–,–,–,–,–,–,1,1,1,1,1,–,–,–,–,–]

where the protein of node 2 is directly activated in the first 180 time-steps (3×60) of the simulation and then protein 1 is indirectly inhibited between time-steps 600 and 900 of the 1200 time-step simulation.

1.2.3 Example of encoding binary strings

As discussed in Fig 1, a section of the 'Promoter' region encodes the logic with which the mRNA of the node is regulated. However, the other regions of the binary string also encode important information. Whilst, in practice, the binary genes can contain a wider range of information (as discussed above), we hope this more detailed example is illustrative of how nodes are encoded. In this example, we highlight a node that is regulated by a transcription factor and goes on to form a protein product that is made of multiple subunits and can dimerise. Each of the respective coding regions (red = mRNA regulation; blue = protein function) represents a different reaction from the dictionaries outlined above. For example, the red regions will tell us whether the mRNA is inhibited or activated by a TF that binds to the mRNA using a particular type of logic. Furthermore, the blue regions tell us

what the function of the dimerised protein product is within the larger network. It is important to note that the order of these regions does not matter, as long as each reaction is contained within the reaction dictionaries such that they can be related to other components in the network and ODEs can be formed from the interactions.

1.3 Basic instructions to create a new EA study

One of the advantages of our EA over previously published variants is that, we believe, ours is general enough that it can tackle many biological problems. Here, we describe the stages and considerations required to use the EA for new tasks.

1. *main.py*
    - Here, the gene pool needs to be altered to allow for any new genes one wishes to allow in their system (e.g. proteins that can respond to external inputs, new complexes, etc.).
    - Check parameter ranges and units.
    - Alter the input vector, if desired, to incorporate multiple different inputs.
2. *graph.py*
    - If new system components are added in *main.py* then new rules need to be included here to allow for these components to be incorporated into evolved networks.
    - In this file the initial networks within the population can also be predefined.
3. *reactions.py*
    - New reactions can be added or removed in this file.
    - For example, if one wished to only include post-translational dynamics then mRNA-based reactions can be removed.
4. *build.py*
    - Set initial conditions of the networks if they are known for specific components.
5. *solve.py*
    - Sort initial conditions so that the pre-defined component initial conditions correspond to the correct model components.
    - Change simulation time-span.

- Edit simulation loop to allow for multiple input conditions.

6. *score.py*

   - Add scoring function.

7. *setup.py*

   - In this file, one can print the ODE equations, the model components, or the reaction list to ensure that the algorithm is functioning correctly.
   - We believe this is important during testing of new EA tasks to ensure accuracy of the resulting analysis.

1.4 Possible extensions and generalisations

The EA can be adjusted to suit the user's needs. This includes altering the reactions that can take place in a network, the manner in which the subsequent models are solved and the manner in which the resulting data is subsequently scored. Altering the EA requires intermediate to advanced programming skills in Python, following the schema structure described in the 'Methods' section.

1.4.1 Reaction library

The reaction library can be extended to include any reaction or set of reactions. In order to achieve this one needs include an element in the subsection of a binary gene that encodes for a rule (Supplementary Fig 1 & 9). We maintained a simple structure regarding protein interactions (dimerisation, subunit formation etc.), however we can consider larger structures such as protein pumps. Protein pumps often consist of multiple components that are either directly or indirectly regulated by an environmental inducer leading to downstream signalling [1]. Importantly, this forms a feed-forward loop that limits the production of protein pump components at the membrane, thereby preventing toxic effects (Supplementary Fig 9) [1]. The pump subsequently removes the inducer from the cell. Suppose we want the EA to build a network responsible for the removal of this inducer after activating the protein pump, we are required to include the appropriate reactions and rules within our libraries to allow for this. Rules for such a reaction would include the necessity of an external inducer to regulate a protein pump inhibitor and the protein pump directly. The external inducer is, in turn, removed from the cell by the protein pump, creating a feedback response.

To incorporate such a reaction within our EA, we need to add a structure in the open reading frame of the binary gene with the rules that place the protein pump in a reaction channel (Supplementary Fig 9, right). There is a degree of freedom

regarding the manner in which the reaction channels are described (e.g. gene activation, repression of a repressor etc.) and assembled. For example, protein pumps could consist of multiple subunits that bind sequentially or two larger subunits that are combined to form the final product. To illustrate this consider the following schematic (Supplementary Fig 9, left). The figure shows an inducer (*i*) that activates a protein (Gene 1) and the production of protein pump components (Gene 2). However, Gene 1 prevents the overexpression of protein pump components that lead to cell death. The rules in the binary genes have been expanded to include protein pumps and their activation by external inducers. Thus, a unique reaction channel is created capable of translating these rules into a set of reaction equations. The result would be the formation of a complex between an inducer with the pump that ultimately leads to the inducer being recycled outside of the cell.

1.4.2 Scoring

Objective functions are the most crucial part of the EA. Therefore we attempted to give the user as much freedom as possible to adjust these functions. The standard scoring function takes as an input the current substrate of the node whose concentration profile the user wishes to score (chosen in the main file as the variable output) and the concentration profile as raw data taken from the solver function. The user can subsequently manipulate this raw data as they see fit to obtain a score. Note that the score serves as an input into the rank based selection of individuals in a generation. In order to streamline this, we suggest all scoring functions minimise an objective. The score or scores (depending on the number of objectives one wishes to have) is subsequently stored in a dictionary and passed to the rank functions.

1.4.3 Solver

All of the results presented in this study have assumed that biological systems can be described deterministically. As discussed in the 'Methods' of the Main Text, the information for each network node is unpacked into ordinary differential equations (ODEs). Consequently, to ensure that the ODEs are appropriately formulated at each iteration of the algorithm, the reaction channels (that list all possible reactions within a system) must be ordered (e.g. Supplementary Fig 1 and Supplementary Fig 9). Without this ordering it is guaranteed that the ODEs are not formulated correctly and the mathematical models do not represent realistic biological scenarios.

The solvers can be adjusted in the *solve.py* file. The possibility exists to solve networks stochastically as well as deterministically. The stochastic solver takes any model generated by the EA and sets up the reactions to be solved using the Gillespie algorithm [2]. The user has to state the number of individual simulations to be performed and the simulation time in this file. Note the Gillespie algorithm we have implemented is able to calculate 30,000 reactions per second. For larger sys-

tems (with larger final times) this means that it's usage becomes infeasible from a computational perspective. This option is therefore better suited as a post analysis step (i.e. after the optimised network is obtained). The file *output.py* can therefore be used. It calls the Gillespie solver and is capable of solving the obtained networks.

1.4.4 Evolutionary Tracking

One of the advantages of using binary strings, matrices and vectors to describe system dynamics is that changes through evolution can be easily tracked. As discussed in the 'Methods', the flipping of a certain bit could lead to an edge deletion in the network, or change the way in which the mRNA of a node is regulated. In this work we have not looked to develop methods of viewing evolutionary changes in network structure, however recent work suggests that current methods could be adapted for these needs [3, 4]. In these works, phylogenetic trees are redrawn as 'split networks' that show how related groups are formed through evolution. McGrane & Charleston show that nodes within the split networks can be described based on the mutation of interaction networks, such as new edges or duplicated nodes [4]. This information is contained within the adjacency matrices of our models. One could envision that using split networks produces a visualisation for how the EA produces desirable networks from a random or known initial system structure. We will look to incorporate such visualisation in future work.

2 Simulation Tests Performed in Main Text

In order to look at the effect of different algorithm settings on computational time and results we performed two benchmarking tests:

1. We perform the EA with a population of 10 networks to find networks of any size that produce oscillations within a 6000 seconds time period. The duration of time required to simulate a single generation (i.e. the 10 individuals) is then recorded. This gives us some idea as to how computational time increases with network complexity.

2. We randomly select algorithm parameters such as

   - population size,
   - mutation frequency,
   - number of (parameter and network) mutations performed each generation,
   - the selection method (proportional, semi-proportional, or elite),

   for 1933 independent EA runs. From the results we then compute a convergence score

   $$\Delta_C = \frac{\Delta_{\text{final}}}{\Delta_{\text{initial}}}. \quad (1)$$

   Here, Δ_{final} and Δ_{initial} are the scores Δ for the fittest individual in the population in the final and initial generations (for details of all scoring functions see the Supplementary Information). We then check for linear regression correlations between Δ_C and the algorithm parameter to determine which aspects of the EA the results are sensitive towards.

Upon completion of these tests we then selected a set of system parameters to perform parameter (fixed network sizes & topologies) and network optimisation to find systems that could produce

1. the concentration profiles (red lines) of Figure 6A—C (simulated time-period = 6000 sec),

2. Repressilator networks (7200 sec),

3. robust oscillating systems using single- and multi-objective functions (4500 sec),

4. networks that respond to input signals, like feed-forward loops (7200 sec).

3 Scoring Functions

To evaluate the fitness of the individual networks within the EA, we need to compare simulations with a desired response. In the main text we discuss the cases of matching concentration profiles, obtaining oscillations using both single- and multi-objective criteria and how oscillating mechanisms can be checked for robustness to internal fluctuations and, below, we discuss the analysis of feed-forward motifs. Here, we shall provide details for each of the scoring functions used.

3.1 Concentration profiles

To compare output simulations to time-series data, we constructed two scoring functions to calculate the score, $\Delta = \delta_1 + \delta_2$, whereby a perfect match implies $\Delta = 0$ (a minimisation problem).

The first of these is the squared residual errors between simulations, y^i_j, and data, d^i_j, for a subset of the nodes, $m \leq M$, in the network

$$\delta_1 = \sum_{i=1}^{m} \sum_{j=1}^{t_p} (d^i_j - y^i_j)^2, \tag{2}$$

where the difference is calculated over the t_p simulated time-points.

The second scoring function used is

$$\delta_2 = \sum_{i=1}^{m} \sum_{j=2}^{t_p} \left(\frac{\Delta d^i_j}{\Delta t} - \frac{\Delta y^i_j}{\Delta t} \right)^2, \tag{3}$$

where $\Delta x / \Delta t$ is the change in x ($\Delta x^i_j = x^i_j - x^i_{j-1}$) over a time-step Δt. We found that incorporating δ_2 helped improve the optimisation of complex time-series dynamics.

3.2 Oscillations

When we evolve networks to find systems that sustain oscillations, we use the scoring function from [5],

$$\Delta = 20 - 2 \sum_{i=1}^{10} \frac{|a_i - a_{i+1}|}{a_i + a_{i+1}} \min(1, |a_i - a_{i+1}|), \tag{4}$$

where the a_i's represent the first 10 extrema (minima or maxima) of an oscillating simulation. Thus, $\Delta = 20$ implies no oscillation and $\Delta = 0$ implies a perfect oscillation with 5 periods during the simulated time-frame [5]. Notably, van Dorp *et*

al. found that strong oscillations could be observed when $\Delta = 4$. This is reflective of the fact that this scoring function can only achieve 0 if the oscillator (and it's reaction rates) exists on a specific limit cycle from the initial time-point. Thus, the scoring function does not account for transitions towards limit cycle behaviour or damping which increase the score away from 0 even if desired oscillations are still present within the simulated time period. To determine whether oscillations show limit cycle behaviour or not, we calculate eigenvalues from the systems Jacobian matrix to assess the networks Hopf bifurcation.

To perform multi-objective optimisation for oscillations we use three objectives

$$\begin{aligned}
\delta_1 &= \frac{1}{P(\omega_{max})}, \\
\delta_2 &= \frac{1}{\omega_{max}}, \\
\delta_3 &= \frac{\int_0^\infty P(\omega)d\omega}{P(\omega_{max})},
\end{aligned} \tag{5}$$

where $P(\omega)$ is the power spectrum estimated from the magnitude of the discrete Fourier Transform of an oscillating concentration and ω_{max} is the frequency at which $P(\omega)$ is greatest. Here, $P(\omega)$ is estimated via

$$P(\omega) = |x_F(\omega)|^2 = \left| \sum_{t=0}^{\infty} x(t)e^{-i\omega t} \right|^2$$

with $x(t)$ being the oscillating signal and $x_F(\omega)$ the Fourier transform [6].

To improve the calculation of the Fourier transform from stochastic simulations, the oscillating signal $x(t)$ is initially smoothed using the Savitzky-Golay filter [7]. Each of δ_1, δ_2, and δ_3 is minimised such that: $\delta_1 \to 0$ implies higher amplitude oscillations, $\delta_2 \to 0$ decreases the period of oscillations (increased frequency), and as $\delta_3 \to 0$ the width of the power spectra decreases implying that the concentration profiles are closer to idealised sine waves.

Furthermore, we also explore the influence of weighting these scores such that some features (e.g. frequency) are favoured over others (e.g. amplitude) within our rank based selection (see the Main Text).

3.3 Robustness

One important factor when creating oscillating networks is to understand the robustness of their behaviour, as has been explored previously [5, 8–10]. Woods *et al.*

have recently used a Bayesian framework to analyse the presence of oscillations in stochastic systems and, similar to them, we define robustness as a measure of average performance over all possible parameter perturbations [10]. To highlight how evolving an oscillating system for robustness could take place, we perform the EA using (4) to find an oscillating system and then change the scoring function such that we look to maximise

$$\rho = \frac{P_S}{P_T}, \tag{6}$$

where P_S is the number of parameter perturbations ($k_j \rightarrow k_j(1+\alpha)$ with $\alpha \sim U(-1,1)$) that produce oscillating systems and P_T is the total number of parameter perturbations performed (250 perturbations for each parameter, k_j). Note that to convert this to a similar minimisation problem as before, we aim to minimise $\Delta = 1/\rho$.

3.4 Feed-forward loops

The objective function we employed in the analysis of feed-forward loops (FFLs) is the same as that used in [11].

The sensitivity of an FFL is defined as the relationship between the input signal, x, and the total production of a node of interest, y. Thus, the inverse of sensitivity is

$$\delta_1(\boldsymbol{\theta}, \mathbf{M}) = \frac{2(x(t_f) - x(t_0))}{\int_{t_0}^{t_f} \left|\frac{dy(t|\boldsymbol{\theta},\mathbf{M})}{dt}\right| dt}, \tag{7}$$

where $\boldsymbol{\theta}$ is a parameter set, $\mathbf{M}$ is the model simulated, t_0 is the time at which the input signal begins and t_f is the final time of the input signal. Consequently, $\delta_1 \rightarrow 0$ as the amount of y produced during the input signal's presence increases.

The precision of a feed-forward loops dynamics are determined by the relaxation of the system at the end of an input signal relative to the starting value. Thus, the inverse of precision is

$$\delta_2(\boldsymbol{\theta}, \mathbf{M}) = \frac{y(t_f|\boldsymbol{\theta},\mathbf{M}) - y(t_0|\boldsymbol{\theta},\mathbf{M})}{x(t_f) - x(t_0)}, \tag{8}$$

where $\delta_2 \rightarrow 0$ as $y(t_f|\boldsymbol{\theta},\mathbf{M}) \rightarrow y(t_0|\boldsymbol{\theta},\mathbf{M})$ and an increase in precision is observed.

As noted by [11], a further constraint is required such that the concentration of y is not too low. Without such a constraint, a large area of parameter space would correspond to high levels of precision and low levels of sensitivity. Consequently, we also included these constraints such that

$$\int_{t_0}^{t_f} \left| \frac{dy(t|\boldsymbol{\theta}, \mathbf{M})}{dt} \right| dt > 1,$$
$$\exists\, \hat{y} \in [y(t_0), y(t_f)] \text{ s.t. } \left\{ \hat{y} > y(t_0), \hat{y} > y(t_f), \left.\frac{dy}{dt}\right|_{y=\hat{y}} = 0 \right\}. \quad (9)$$

These constraints essentially ensure the presence of a peak within the time simulations and that the total concentration of y during the presence of input signal, x, is high.

4 Multi-objective analysis of feed-forward networks

The abundance of feed-forward network motifs in biological systems was first noted by the group of Uri Alon [12–14]. The motif is made up of 3 nodes (X, Y, and Z) whereby X regulates both Y and Z, with Y also regulating Z. The FFL family consists of 8 members: 4 coherent loops whereby Z is regulated either positively or negatively by both X-dependent pathways, and 4 incoherent members whereby Z is regulated in an opposite manner by X through the two paths [13]. These motifs have been shown to act as signal transducers in response to input signals, responding either quickly or slowly to the signal depending on the motif structure. Recently, the type-1 incoherent FFL has been analysed using multi-objective optimisation to find parameter sets that either (1) respond quickly to an input signal, or (2) relaxes precisely back to the pre-input dynamics after the signal-induced response [11]. This work highlighted that tuning the production rates of Y, plus the dynamics of Z can lead to an type-1 incoherent FFL switching from a sensitive to a precise response and *vice versa* along the Pareto front. Here, we shall provide the details of the objective function used in [11], show that our multi-objective EA is able to provide similar results before generalising the approach to analyse different logic-gates within the FFLs and the effect of different input signals.

4.1 Results

4.1.1 Type-1 incoherent feed-forward loops

In the main text (Figure 11), we show the Pareto front obtained for type-1 incoherent FFLs that are scored for sensitivity to input signals and their precision in returning to their pre-input state. As further analysis we looked at the optimal parameter values for precise and sensitive FFLs (Supplementary Table 10). By examining which parameters were significantly different (p-value < 0.05), we found four parameters that help determine whether a system responds sensitively or precisely — k_3, k_9, k_{23}, k_{24}. These correspond to

- k_3 = binding of inducer molecule to protein X,
- k_9 = degradation of X when not induced,
- k_{23} = degradation of Z,
- k_{24} = unbinding rate of inducer molecule with X.

The importance of degradation rates of system components and the production of Y and Z (that are regulated by an induced form of X) in the incoherent feed-forward

loop has been noted previously in [11]. Due to the qualitatively similar results with previous observations seen in Figure 11, we now look at a more general case where all feed-forward loops are evolved.

4.1.2 All feed-forward loops

As well as the FFL motifs having different connections between components, one can also alter the logic with which components are regulated. This results in a much larger number of networks within network space and properties that can be explored. We used our EA to obtain Pareto fronts in four conditions:

1. The input signal on X is continuous and:
   - components are regulated by cofactor TFs.
   - components are regulated by competitive TFs.
2. The input signal on X is pulsed and:
   - components are regulated by cofactor TFs.
   - components are regulated by competitive TFs.

The Pareto fronts for each condition can be observed in Supplementary Fig 7. Interestingly the results show that only a half of the possible FFL motifs are favoured when optimised for precision and sensitivity — incoherent FFLs type-1 & type-2 and coherent FFLs type-3 & type-4. Notably, given the four conditions tested we make the following observations:

- incoherent FFLs type-1 are favoured for:
  - high sensitivity and precision given competitive TFs and a continuous signal is present.
  - high precision and low sensitivity given pulsed signal inputs for either regulatory form.
- incoherent FFLs type-2 are favoured for:
  - high precision and low sensitivity given competitive TFs and a continuous signal.
  - high sensitivity but relatively low precision given pulsed inputs for either regulatory form.
- coherent FFLs type-3 are favoured for:
  - moderate sensitivity and precision given competitive TFs and a continuous signal.

  - moderate sensitivity and precision given either regulatory form and a pulsed signal.

- coherent FFLs type-4 are favoured for:

  - all levels of sensitivity and precision given cofactor TFs and a continuous signal.

Based on these results, we make two observations. First, for the purposes of synthetic design only half of the FFL motifs provide optimal or tunable responses along the Pareto front, suggesting that other FFL motifs should not be considered for applications. Second, given that incoherent FFLs type-1 and type-2 are able to obtain either optimal precision or sensitivity, it is interesting to note that only the incoherent FFL type-1 is found commonly in nature [13]. This could suggest that species have optimised their response to input signals such that there is high precision rather than high sensitivity. Conversely, the reason that coherent FFL type-3 and type-4 are relatively rare in biology could be due to their tunability, i.e. biological systems favour motifs that provide one optimal and desired response to input signals rather than a motif that is flexible. Whilst this final conclusion is speculative, we believe that our analysis provides a more general framework for understanding FFL properties to previous observations.

References

[1] M. J. Dunlop, J. D. Keasling, and A. Mukhopadhyay. A model for improving microbial biofuel production using a synthetic feedback loop. *Systems and Synthetic Biology*, 4:95–104, 2010.

[2] D. T. Gillespie. Stochastic simulation of chemical kinetics. *Annual Review of Physical Chemistry*, 58:35–55, 2007.

[3] D. H. Huson and D. Bryant. Application of phylogenetic networks in evolutionary studies. *Molecular Biology and Evolution*, 23:254–267, 2006.

[4] M. McGrane and M. A. Charleston. Biological network edit distance. *Journal of Computational Biology*, 23:DOI: 10.1089/cmb.2016.0062, 2016.

[5] M. van Dorp, B. Lannoo, and E. Carlon. Generation of oscillating gene regulatory network motifs. *Physical Review E*, 88:012722, 2013.

[6] J. W. Cooley and J. W. Tukey. An algorithm for the machine calculation of complex fourier series. *Mathematics of Computation*, 19:297–301, 1965.

[7] A. Savitzky and M. J. E. Golay. Smoothing and differentiation of data by simplified least squares procedures. *Analytical Chemistry*, 36:1627–1639, 1964.

[8] T. Y. C. Tsai, Y. S. Choi, W. Ma, J. R. Pomerening, C. Tang, and J. E. Ferrell. Robust, tunable biological oscillations from interlinked positive and negative feedback loops. *Science*, 321:126–129, 2008.

[9] Y. C. Chang, C. L. Lin, and T. Jennawasin. Design of synthetic genetic oscillators using evolutionary optimization. *Evolutionary Bioinformatics*, 9:137–150, 2013.

[10] M. L. Woods, M. Leon, R. Perez-Carrasco, and C. P. Barnes. A statistical approach reveals designs for the most robust stochastic gene oscillators. *ACS Synthetic Biology*, 5:459–470, 2016.

[11] Y. Boada, G. Reynoso-Meza, J. Pico, and A. Vignoni. Multi-objective optimization framework to obtain model-based guidelines for tuning biological synthetic devices: an adaptive network case. *BMC Systems Biology*, 10:27, 2016.

[12] R. Milo, S. Shen-Orr, S. Itzkovitz, N. Kashtan, D. Chklovskii, and U. Alon. Network motifs: Simple building blocks of complex networks. *Science*, 298:824–827, 2002.

[13] S. Mangan and U. Alon. Structure and function of the feed-forward loop network motif. *Proceedings of the National Academy of Sciences USA*, 100:11980–11985, 2003.

[14] N. Kashtan and U. Alon. Spontaneous evolution of modularity and network motifs. *Proceedings of the National Academy of Sciences USA*, 102:13773–13778, 2005.

Supplementary Tables

S1 Table: Encoding TF binding to promoters. α and β refer to binary sections of the gene string (Fig 1).

Promoter \ TF	α	β
α	Activation	Inhibition
β	Inhibition	Activation

S2 Table: Reactions behind promoter regulation of nodes.

**Cofactor TFs**		
Promoter + TF (1) + TF (2)	→	Promoter-TF-TF
Promoter-TF-TF	→	Promoter + TF (1) + TF (2)
Promoter-TF-TF	→	Promoter-TF-TF + mRNA
**Competitive TFs**		
Promoter + TF (1)	→	Promoter-TF (1)
Promoter-TF (1)	→	Promoter + TF (1)
Promoter + TF (2)	→	Promoter-TF (2)
Promoter-TF (2)	→	Promoter + TF (2)
Promoter-TF (1) + TF (2)	→	Promoter-TF-TF
Promoter-TF (2) + TF (1)	→	Promoter-TF-TF
Promoter-TF-TF	→	Promoter-TF (2) + TF (1)
Promoter-TF-TF	→	Promoter-TF (1) + TF (2)
Promoter-TF (1)	→	Promoter-TF (1) + mRNA
Promoter-TF (2)	→	Promoter-TF (2) + mRNA
Promoter-TF-TF	→	Promoter-TF-TF + mRNA
**Sign-dependent competition**		
Promoter + TF (1)	→	Promoter-TF (1)
Promoter-TF (1)	→	Promoter + TF (1)
Promoter + TF (2)	→	Promoter-TF (2)
Promoter-TF (2)	→	Promoter + TF (2)
Promoter-TF (1)	→	Promoter-TF (1) + mRNA

S3 Table: Protein dimerisation reactions.

**Dimerisation**		
Protein + Protein	$\rightarrow$	Protein-Protein
Protein-Protein	$\rightarrow$	Protein + Protein

S4 Table: Protein regulatory reactions.

**Active Protein Inhibition**		
Active Inhibitor + Target	→	Active Inhibitor-Target Complex
Active Inhibitor-Target Complex	→	Active Inhibitor + Target
Active Inhibitor-Target Complex	→	Active Inhibitor
**Passive Protein Inhibition**		
Passive Inhibitor + Target	→	Passive Inhibitor-Target Complex
Passive Inhibitor -Target Complex	→	Passive Inhibitor + Target
**Active Protein Activator**		
Active Activator + Target	→	Active Activator-Target Complex
Active Activator - Target Complex	→	Active Activator + Target
Active Activator-Target Complex	→	Active Inhibitor + Activated Target
**Passive Protein Activator**		
Passive Activator + Target	→	Passive Activator-Target Complex
Passive Activator - Target Complex	→	Passive Activator + Target

S5 Table: Protein switching reactions.

**Direct Activation**		
Product	$\rightarrow$	activated-product
Activated-Product	$\rightarrow$	Product
**Indirect Activation**		
Product + Inducer	$\rightarrow$	Product-Inducer
Product-Inducer	$\rightarrow$	Product + Inducer
Inducer	$\rightarrow$	Ø
**Direct Inhibition**		
Product	$\rightarrow$	Deactivated-Product
Deactivated-Product	$\rightarrow$	Product
**Indirect Inhibition**		
Product + Inhibitor	$\rightarrow$	Product-Inhibitor
Product-Inhibitor	$\rightarrow$	Product
Inhibitor	$\rightarrow$	Ø

S6 Table: Subunit formation of protein.

**Subunit Binding**		
Gene + Polymerase	$\rightarrow$	Gene + Polymerase + mRNA (1) + mRNA (2) + mRNA (3)
mRNA (1) + Ribosome	$\rightarrow$	mRNA (1) + Ribosome + Protein (1)
mRNA (2) + Ribosome	$\rightarrow$	mRNA (2) + Ribosome + Protein (2)
mRNA (3) + Ribosome	$\rightarrow$	mRNA (3) + Ribosome + Protein (3)
Protein (1) + Protein (2)	$\rightarrow$	Protein-Protein
Protein-Protein + Protein(3)	$\rightarrow$	Protein-Protein-Protein

S7 Table: Default parameter ranges.

Reaction Type	Reaction Rate Boundaries
Transcription	0.1-12
Translation	0.1-10
Protein-Protein binding	10-100
Gene-Protein binding	10-100
Protein activation	Forward 10-1000, Reverse 0.1-10
Gene activation	10-25
Protein catalysis	10-1000
mRNA degradation	0.3-0.99
Protein degradation	0.01-0.99
Transport	0.1-100

S8 Table: Default mutation rates.

Mutation	Probability
Mutate Binary Gene	0.40
Add Binary Gene	0.10
Delete Binary Gene	0.15
Add Connection	0.10
Delete Connection	0.15
Move Connection	0.10

S9 Table: Parameter definitions within the Repressilator of Fig 11E.

Parameter	Name	Definition
k_1	'g0m0GR_Rg0m0GR_Rk_dim'	dimerisation protein 0 protein 0
k_2	'g1m0GR_Rg1m0GR_Rk_dis'	dissociation protein 1 protein 1
k_3	'g2m0GR_Rg2m0GR_Rk_dis'	dissociation protein 2 protein 2
k_4	'g1g0m0GR_Rg0m0GR_RkG_A_bnd'	binding dimer 0 to gene 1
k_5	'g1m0kM_deg'	degradation mRNA 1
k_6	'g2m0GR_Rg2m0GR_Rk_dim'	dimerisation protein 2 protein 2
k_7	'g2m0k_trns'	translation mRNA 2
k_8	'g0m0k_trns'	translation mRNA 0
k_9	'g1m0GR_RkP_deg'	degradation protein 1
k_{10}	'g2m0kM_deg'	degradation mRNA 2
k_{11}	'g1g0m0GR_Rg0m0GR_RkG_A_unbnd'	unbinding dimer 0 gene 1
k_{12}	'g1m0GR_Rg1m0GR_Rk_dim'	dimerisation protein 1 protein 1
k_{13}	'g0k_trcb'	transcription gene 0
k_{14}	'g1k_trcb'	transcription gene 1
k_{15}	'g1m0k_trns'	translation mRNA 1
k_{16}	'g0g2m0GR_Rg2m0GR_RkG_A_unbnd'	unbinding dimer 2 gene 0
k_{17}	'g2m0GR_Rg2m0GR_RkP_deg'	degradation dimer 2
k_{18}	'g2g1m0GR_Rg1m0GR_RkG_A_unbnd'	unbinding dimer 1 gene 2
k_{19}	'g0g2m0GR_Rg2m0GR_RkG_A_bnd'	binding dimer 2 gene 0
k_{20}	'g1m0GR_Rg1m0GR_RkP_deg'	degradation dimer 1
k_{21}	'g2g1m0GR_Rg1m0GR_RkG_A_bnd'	binding dimer 1 gene 2
k_{22}	'g0m0GR_RkP_deg'	degradation protein 0
k_{23}	'g2m0GR_RkP_deg'	degradation protein 2
k_{24}	'g2k_trcb'	transcription gene 2
k_{25}	'g0m0kM_deg'	degradation mRNA 0
k_{26}	'g0m0GR_Rg0m0GR_Rk_dis'	dissociation dimer 0
k_{27}	'g0m0GR_Rg0m0GR_RkP_deg'	degradation dimer 0

S10 Table: Parameter values for sensitive and precise incoherent feed-forward loops.

Parameter	Average (Sensitive)	Standard Error	Average (Precise)	Standard Error	P-value of similarity
k_1	0.73	0.019	0.74	0.03	0.776
k_2	0.811	0.071	0. 797	0.072	0.894
k_3	6.954	0.528	4.665	1.154	0.059
k_4	4.06	0.593	3.895	0.528	0.846
k_5	2.232	0.692	1.695	0.679	0.6
k_6	6.116	0.513	5.325	0.983	0.447
k_7	1.688	0.439	2.067	0.489	0.577
k_8	4.288	0.414	4.72	0.747	0.592
k_9	0.248	0.053	0.442	0.07	0.037
k_{10}	5.355	0.757	4.72	1.004	0.613
k_{11}	0.448	0.054	0.451	0.036	0.962
k_{12}	2.483	0.441	2.984	0.399	0.433
k_{13}	0.661	0.027	0.65	0.036	0.807
k_{14}	0.756	0.053	0.638	0.074	0.197
k_{15}	0.768	0.069	0.613	0.107	0.216
k_{16}	0.684	0.067	0.577	0.067	0.287
k_{17}	0.756	0.074	0.662	0.078	0.406
k_{18}	0.605	0.053	0.451	0.063	0.078
k_{19}	0.3	0.0	0.3	0.0	1
k_{20}	0.739	0.048	0.723	0.06	0.84
k_{21}	4.974	0.595	4.17	0.689	0.391
k_{22}	0.718	0.024	0.652	0.025	0.08
k_{23}	0.254	0.043	0.416	0.058	0.034
k_{24}	2.27	0.467	4.06	0.644	0.032

Supplementary Figures

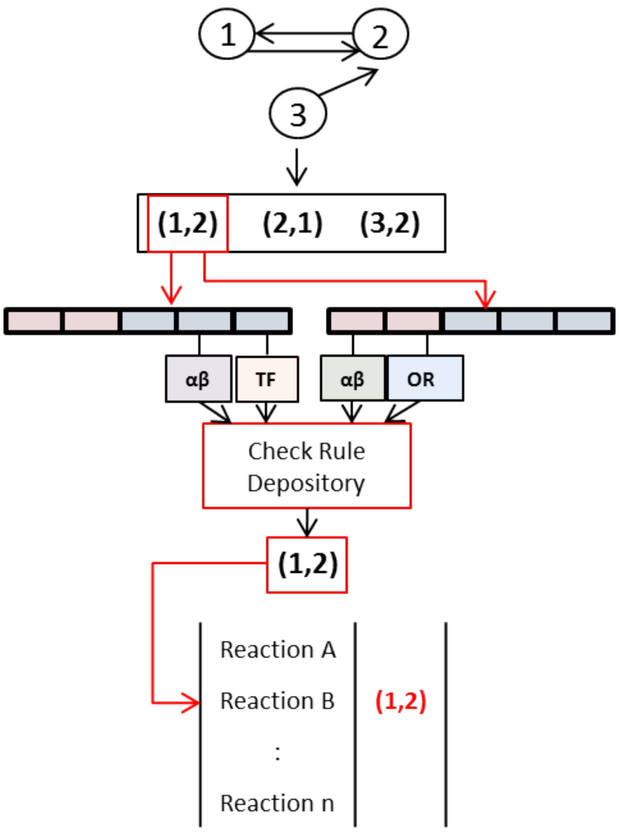

S1 Fig: A conceptual overview as to how reactions are ordered. From the adjacency matrix of the network we know that Nodes 1 and 2 regulate each other and that Node 3 is regulated by Node 2. The binary strings of Nodes 1 and 2 tell us that Node 1 regulates the mRNA production of Node 2 via an OR gate as the protein sequence and function of Node 1 is matched to the promoter sequence of Node 2 (Supplementary Table 1). The rules are checked against a reaction library. Each reaction is then ordered to be unpacked sequentially into the networks reaction scheme (Eq (1) of the main text).

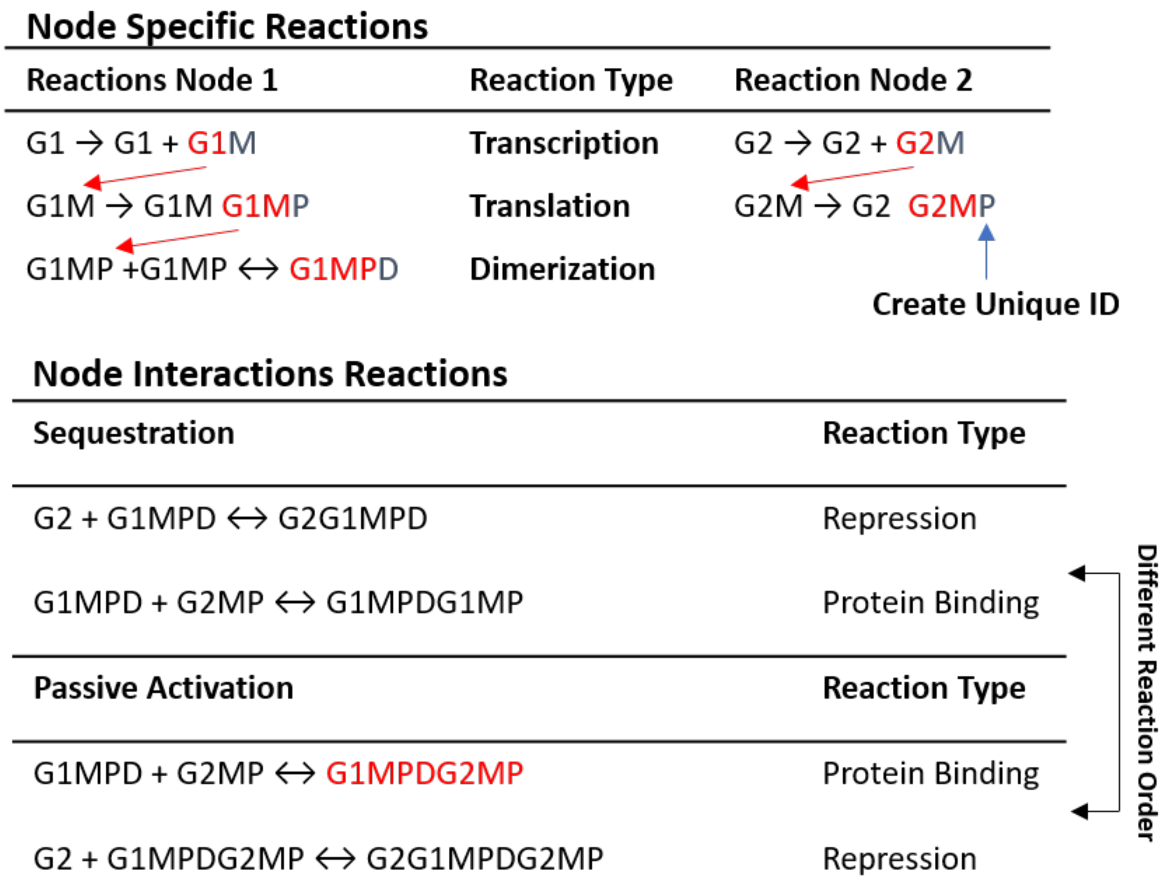

S2 Fig: Example of how reaction IDs are created. Reactions are labelled through the use of IDs. These IDs relate to the order of reactions taking place. Both node-specific and interacting reactions are encoded by specific IDs. Changing the order of the reactions leads to unique IDs being created and altered system dynamics. The ID structure makes it easier to track system-wide changes during the evolutionary process.

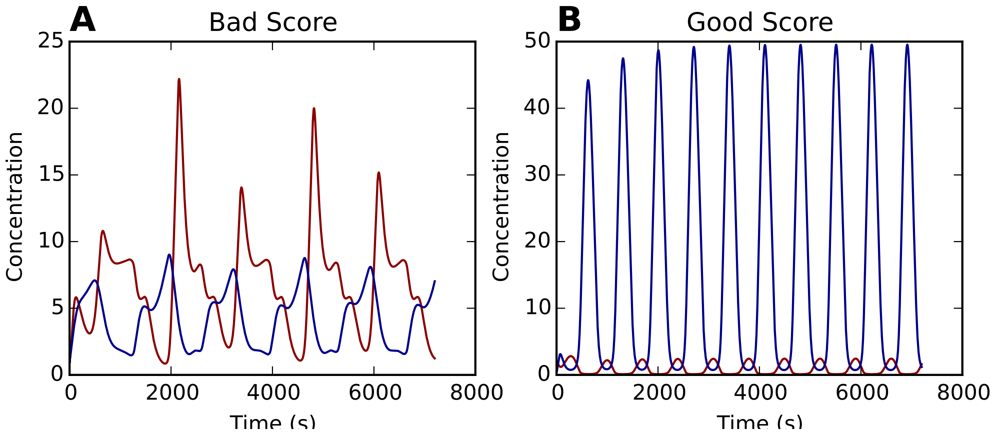

S3 Fig: Time-series of optimal oscillating networks in Fig 7. (A) Time-series of an optimal network that has a subcritical Hopf bifurcation and (B) stable Hopf bifurcation.

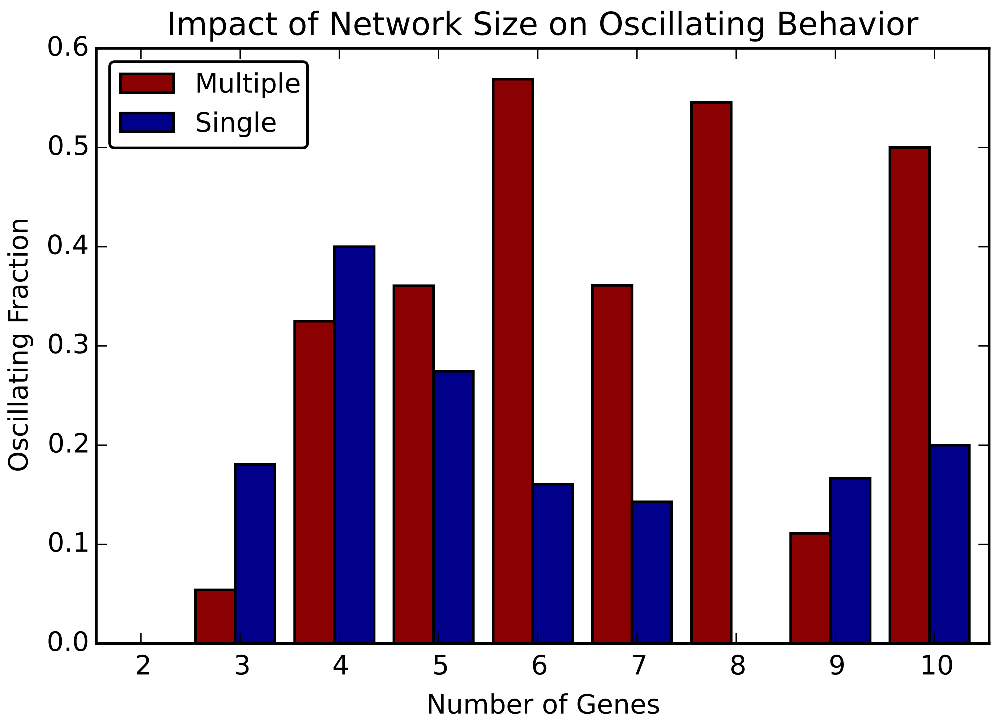

S4 Fig: Distribution of network sizes for networks with stable oscillations. The distribution for network sizes is shown for networks with stable oscillations whereby TFs have a (blue) single activation/inhibitory function or (red) both functions.

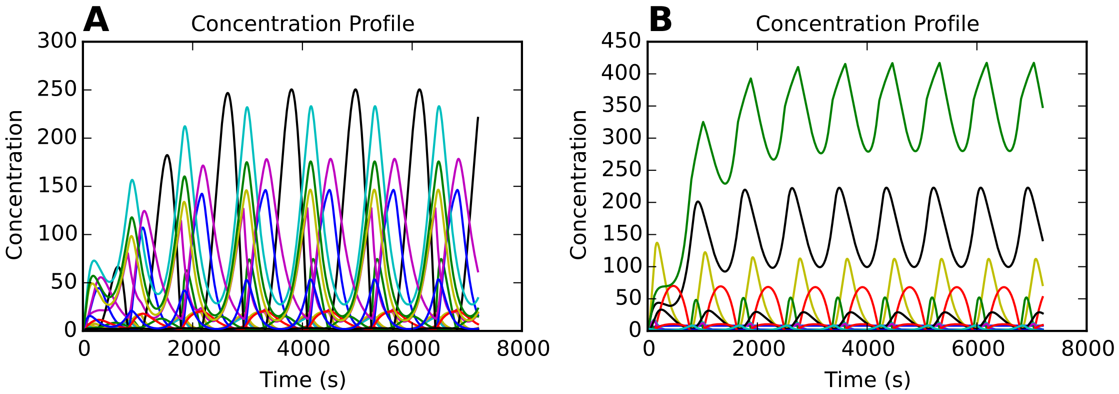

S5 Fig: Deterministic simulations of oscillators. Simulations of network components from (A) the most robust oscillator and (B) most sensitive network.

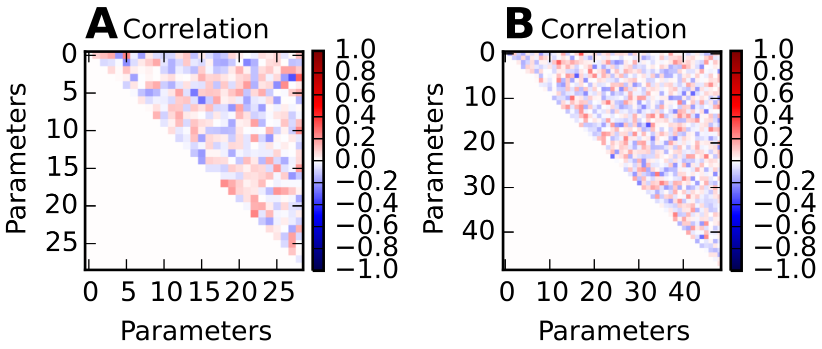

S6 Fig: Pairwise parameter correlations for robust networks. Two examples of optimally robust networks and their pairwise correlation to the objective score.

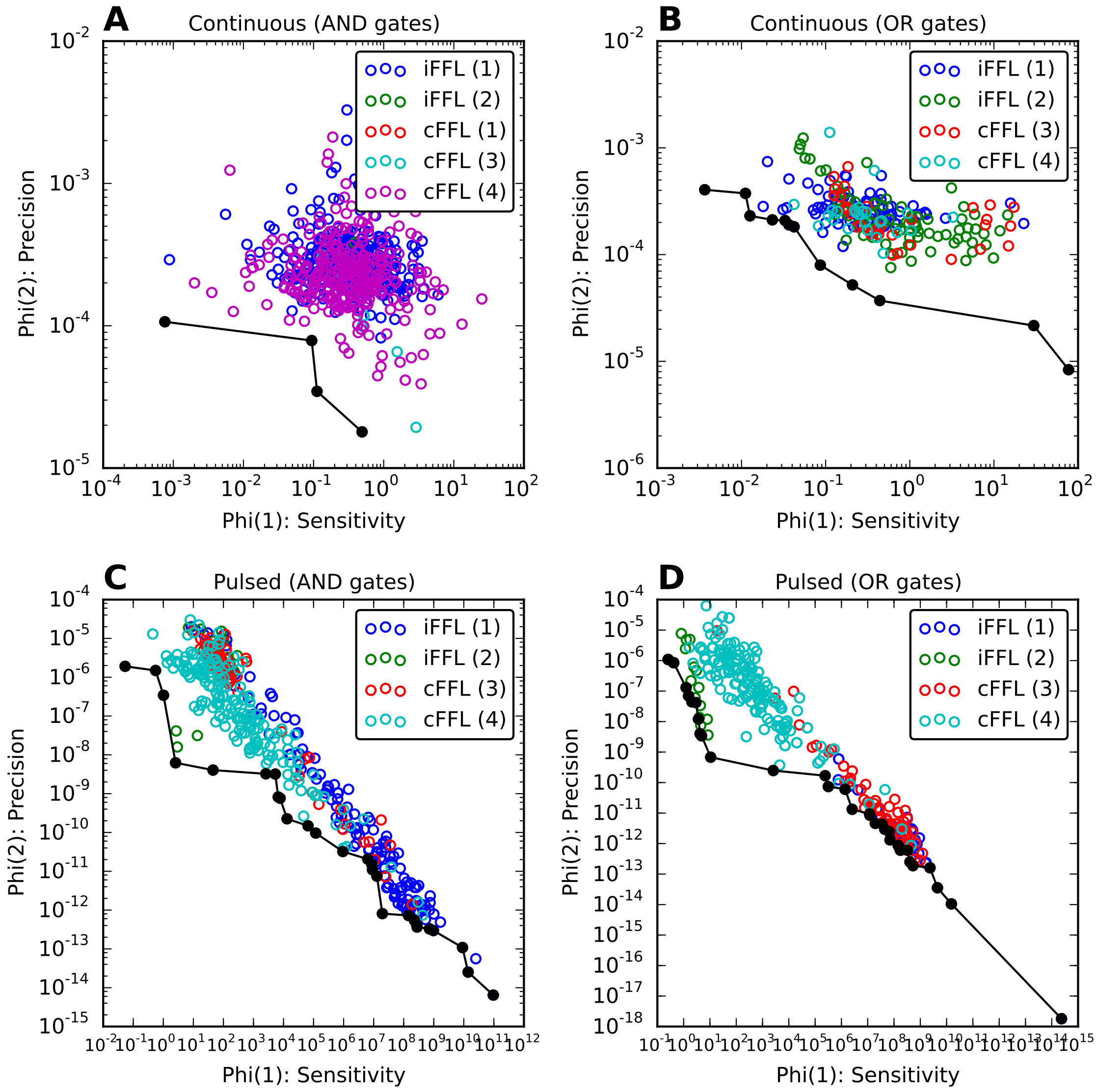

S7 Fig: Pareto front for all feed-forward loops under different conditions. FFLs containing (A, C) AND-gates or (B, D) OR-gates are subjected to a (A, B) constant input signal or (C, D) pulsed input.

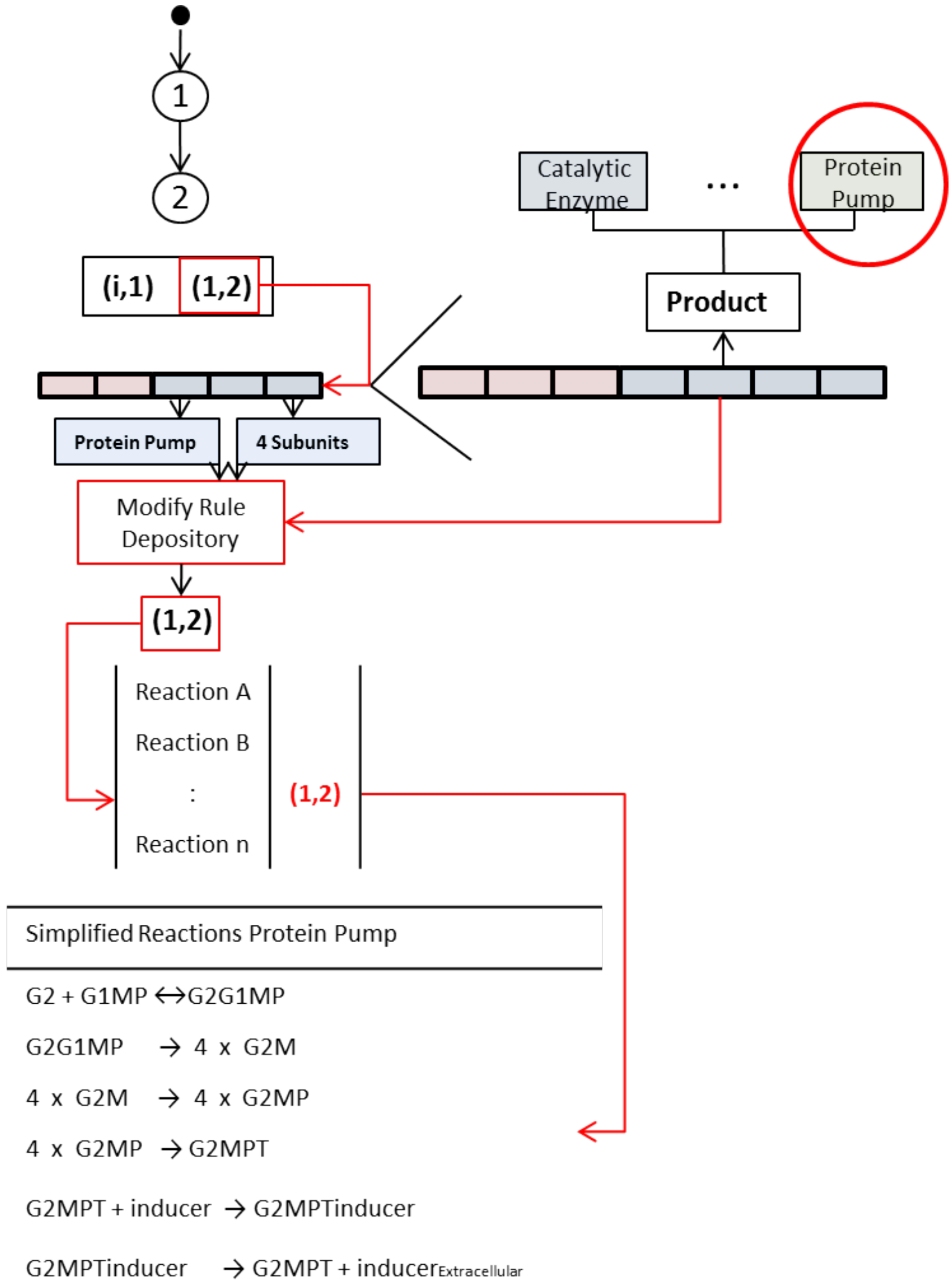

S8 Fig: Example of encoding a protein pump within the reaction library. To encode a protein pump both the reaction library and the node schema need to be updated to allow for the required interactions. This leads to new reaction channels being encoded to allow for protein pump formation.
